# The Applicability of 2-amino-4,6-diphenyl-pyridine-3-carbonitrile Sensors for Monitoring Different Types of Photopolymerization Processes and Acceleration of Cationic and Free-Radical Photopolymerization Under Near UV Light

**DOI:** 10.3390/s19071668

**Published:** 2019-04-08

**Authors:** Joanna Ortyl, Paweł Fiedor, Anna Chachaj-Brekiesz, Maciej Pilch, Emilia Hola, Mariusz Galek

**Affiliations:** 1 Faculty of Chemical Engineering and Technology, Cracow University of Technology, Warszawska 24, 31-155 Cracow, Poland; pfiedor@indy.chemia.pk.edu.pl (P.F.); pilchmac@gmail.com (M.P.); emiliahola@chemia.pk.edu.pl (E.H.); 2 Photo HiTech Ltd., Bobrzyńskiego 14, 30-348 Cracow, Poland; mariusz.galek@photohitech.com; 3 Faculty of Chemistry, Jagiellonian University, Gronostajowa 2, 30-387 Cracow, Poland; chachaj@chemia.uj.edu.pl

**Keywords:** fluorescent molecular sensors, luminescence spectroscopy, photopolymerization

## Abstract

The performance of a series of 2-amino-4,6-diphenyl-pyridine-3-carbonitrile derivatives as fluorescent molecular sensors for monitoring photopolymerization processes of different monomers by the Fluorescence Probe Technique (FPT) was studied. It has been shown that the new derivatives are characterized by much higher sensitivity than the commercially available 7-diethylamino-4-methylcoumarin (Coumarin 1) and trans-2-(2′,5′-dimethoxyphenyl)ethenyl-2,3,4, 5,6-pentafluorobenzene (25ST) probes. It has been discovered that the 2-amino-4,6-diphenyl-pyridine-3-carbonitrile derivatives accelerate the cationic photopolymerization process initiated with diphenyliodonium photoinitiators at the wavelength where the photoinitiator alone does not work. They are particularly efficient for the photoinitiation of cationic photopolymerization of an epoxide and vinyl monomers. Consequently, the application of the 2-amino-4,6-diphenyl-pyridine-3-carbonitrile derivatives in a dual role: (a) as fluorescent sensors for monitoring the free-radical, thiol-ene and cationic polymerization progress, and (b) as long-wavelength co-initiators for diphenyliodonium salts initiators, is proposed.

## 1. Introduction

Fluorescence spectroscopy is an important analytical technique that has been widely used in a variety of applications, such as biomedicine, biology, and science, which makes it unique thanks to its extraordinary sensitivity and selectivity, short delay time (<10^−9^ s), and the fact that it is neither invasive nor destructive, so it can be used for the in-situ measurements [1]. However, the incorporation of additives with the fluorescent chromophores is necessary. When the fluorescence emission of these molecules is sensitive to changes of properties, they can be used for detecting changes in their microenvironment, and in this case, they are called luminescent or fluorescent sensors or probes [2,3].

Fluorescent molecular sensors, i.e., substances capable of emission of the fluorescent light after excitation by light of a certain length, find their applications everywhere where the possibilities of conventional measurement apparatus expire. They allow monitoring of parameters of chemical and physical processes in the real time [4,5]. Due to molecular sizes of the sensor, information obtained through it mainly concerns its closest surroundings. The chemical composition of the molecular sensor can display sensitivity to changes in polarity [6,7], viscosity [8,9,10], temperature [11,12,13], pressure [14,15], oxygen [16] and other parameters of the researched system. To monitor the observed process, the parameters are explicitly used for the applied molecular probe, such as, for example, changes in the character of the absorption spectrum [17] or fluorescence [18,19]. Nowadays the method of fluorescent molecular sensors finds extensive use in different areas of science, starting with molecular biology [20,21] to chemistry of polymers [22,23]. Undoubtedly, it is currently the fundamental research tool in the molecular biology [24,25], where it serves to mark the presence of ions [26,27], hormones [28] or other chemical individuals. Hence, the fluorescent sensors are a unique and quick diagnostic tool with remarkably high sensitivity. Fluorescent sensors that are broadly used are introduced into preparations to specifically visualize specific sub-cell structures or some of the physiological processes using fluorescence microscopes [29]. Nevertheless, using fluorescent sensors is not limited only to the areas of life science; namely, appropriately selected molecular sensors are used to monitor changes of selected physiochemical parameters in real time in different types of materials. Thanks to this they find use in mapping the distribution of pressure on the surfaces of tested objects in the aerodynamic tunnels [30], contactless temperature measurement [31] and pressure [32]. However, regardless of the field in which the fluorescent sensors are used, the combination of appropriate spectral properties and the required sensitivity to the external signals continues to be the current challenge for the contemporary science.

A completely different direction of using the fluorescent sensors are the studies relates to monitoring and control of the polymerization processes [33,34] and photopolymerization [35,36], as well as the nature of the obtained polymers [37]. The development of photochemical techniques using the molecular fluorescent sensors as a research tool in the polymer chemistry took place along with the development of the Fluorescence Probe Technology (FPT). In the chemistry of polymers, the FPT technology is used mainly within the framework of monitoring the progress of polymerization and photopolymerization processes [38], as well as for optimizing the processes of manufacturing the photocurable polymer coatings [39], and the quantitative assessment of the efficiency of new photoinitiators of polymerization [40,41]. The FPT technique has a number of advantages that traditional methods of control of polymerization progress are devoid of; it is a non-destructive method, with a high sensitivity, characterized by a short response time [42]. Importantly, it can also be used to measure the course of polymerization processes both on-line and in-situ through fluorescent sensors specifically designed for this purpose that display the sensitivity of characteristics of fluorescence spectrum to the changes in their direct microenvironment [43]. This is possible because their fluorescence is sensitive to the polarity and/or microviscosity of the molecular environment in which the sensor molecules are located [44]. This dependence is due to the fact that with the progress of polymerization of the monomer the polarity of the system decreases, as more polar double bonds of monomer are converted into fewer polar single bonds in the polymer. At the same time, the viscosity of the system increases, used in the context of sensors the term of sensitivity to changes of microviscosity relates to the fact of the measurement of the viscosity of the system in relation to the closest microenvironment of the molecular sensor, and not to the macroscopic measurements of viscosity. As a result, with the progress of polymerization, the fluorescence spectrum is shifted towards the shorter waves, which is directly related to the increase of microviscosity and with the progress of polymerization process, as well as the increase of conversion of a monomer. Thus, the fluorescent sensors used in the FPT technique enable the visualization of the progress of photopolymerization process both on-line and in-situ during the process.

To this day, several different types of fluorescent sensors have been developed serving as a tool in the FPT technique to monitor the processes of photopolymerization [45], differing between themselves in terms of operational mechanisms [46,47]. However, depending on the studied environment, a specific composition, sensitivity and quantum yield of the fluorescent probe are required. Therefore, there are no completely universal probes. However, each process causing changes in the polarity or microviscosity of the system can be monitored by the FPT method, but depending on the character of the occurring changes, it requires an appropriately selected probe. For this reason, the majority of currently known molecular probes suitable for monitoring the free radical polymerization [48] are generally not suitable for systems cured according to the cationic mechanism and vice versa. The limited universality of the currently known compounds to use as a probe makes it still necessary to seek new molecules for the role of fluorescent probes for monitoring the processes of cationic, free radical, thiol-ene and hybrid polymerization.

In this paper we describe the performance of a series of 2-amino-4,6-diphenyl-pyridine-3-carbonitrile derivatives as potential fluorescent probes for monitoring of free radical and thiol-ene photopolymerization of monomers, in comparison to 7-diethylamino-4-methylcoumarin, which is a typical probe used for monitoring of free radical polymerization. Compared to 7-diethylamino-4-methylcoumarin the tested 2-amino-4,6-diphenyl-pyridine-3-carbonitrile derivatives show very good sensitivity under free-radical and thiol-ene photopolymerization conditions. Moreover the facile synthesis of these compounds makes them good candidates for application as fluorescent probes for the processes carried out in the polymer industry. A significant feature of these probes is that they exhibit an accelerated effect of the cationic photopolymerization of vinyl ethers and are suitable for the role of co-initiators in the bimolecular photoinitiating system based on iodonium salt upon UV-LED with the emission at 365 nm, which is the main ecologically friendly source of light in the photochemical industry.

## 2. Materials and Methods

### 2.1. Materials

Triethylene glycol divinyl ether (TEGDVE, from Sigma Aldrich) and 3,4-epoxycyclohexylmethyl 3,4-epoxycyclohexanecarboxylate (CADE, from Allnex) were applied as a model vinyl ether monomer and cycloaliphatic epoxide monomer respectively for the compositions cured by cationic photopolymerization. For the role of cationic photoinitiator the diphenyliodonium hexafluorophosphate (HIP, from Alfa Aesar) was used. On the other hand, trimethylopropane triacrylate (TMPTA, from Sigma Aldrich) and 2,2-dimethoxy-2-phenylacetophenone (DMPA, from Sigma Aldrich), were employed as a methacrylate monomer and a free-radical photoinitiator for the compositions polymerized by a free-radical mechanism for FPT experiments. Furthermore, trimethylopropane trimethacrylate (TMPTMA, from Sigma Aldrich) and trimethylopropane tris(3-mercaptopropionate) (MERCAPTO, from Sigma Aldrich) were utilized as a methacrylate and thiol monomers for the compositions polymerized by the thiol-ene mechanism. 2,2-dimethoxy-2-phenylacetophenone (DMPA, from Sigma Aldrich) was applied as the photoinitiator in the thiol-ene polymerization process for FPT experiments.

The series of derivatives of 2-amino-4,6-diphenyl-pyridine-3-carbonitrile were investigated in the role of luminescent molecular sensors. These were the derivatives of 2-amino-4,6-diphenyl-pyridine-3-carbonitrile (S1), 2-amino-4-(4-methoxyphenyl)-6-phenyl- pyridine-3-carbonitrile (S2), 2-amino-6-(4-methoxyphenyl)-4-phenyl-pyridine-3-carbonitrile (S3), 2-amino-4,6-bis(4-methoxyphenyl)pyridine-3-carbonitrile (S4), 2-amino-6-(4-cyanophenyl)-4-(4-methoxyphenyl)pyridine-3-carbonitrile (S5) and 2-amino-4-(4-cyanophenyl)-6-(4-methoxyphenyl)-pyridine-3-carbonitrile (S6). The studied details of synthesis and physicochemical data of the derivatives of 2-amino-4,6-diphenyl-pyridine-3-carbonitrile are given in the Supporting Information. The structures of the compounds are shown in Table 1.

As a reference, the molecular fluorescent sensors for monitoring of the progress of photopolymerization processes by FPT method were employed respectively: the amine-free fluorescent sensor trans-2-(2′,5′-dimethoxyphenyl)ethenyl-2,3,4, 5,6-pentafluorobenzene (25ST) for monitoring of cationic photopolymerization, which was provided by Photo HiTech (Poland), and 7-diethylamino-4-methylcoumarin (Coumarin 1, from Sigma Aldrich) for monitoring of free-radical and thiol-ene photopolymerization processes.

### 2.2. Absorption and Fluorescence Characteristics

As a reference, the molecular Absorption spectra of the photoinitiators were recorded in acetonitrile, using the SilverNova spectrometer (StellarNet, Inc., Tampa, FL, USA) in combination with a broadband tungsten-deuterium UV-Vis light source (from StellarNet, Inc., Tampa, FL, USA), and a quartz cuvette with 1.0 cm optical path. Next, the absorbance data were converted into extinction coefficients, expressed in classical units [dm^3^·mol^−1^·cm^−1^].

Fluorescence measurements were carried out using the same miniature spectrometer. The spectral characteristics of the sensors were measured in acetonitrile at room temperature (22 °C) using 10 mm thick quartz cells. As a source of excitation, the UV-LED 320 nm (UVTOP315-BL-TO39, Roithner Laser Technik GmbH, Wien, Austria) light was used.

### 2.3. Electrochemical Characteristics Determination of Oxidation Potentials

The oxidation potentials (E_ox_ vs. Ag/AgCl) of the studied derivatives of 2-amino-4,6-diphenyl-pyridine-3-carbonitrile were measured in acetonitrile by cyclic voltammetry with tetrabutylammonium hexafluorophosphate (0.1 M) as a supporting electrolyte (Electrochemical Analyzer M161 and the Electrode Stand M164, from MTM-ANKO, Cracow, Poland). The working electrode was a platinum disk and the reference was a silver chloride electrode—Ag/AgCl; a scan rate of 0.1 V/s has been used; ferrocene was used as a standard and the potentials were determined from half peak potentials. The Gibbs free energy change ΔGet for an electron transfer between photoinitiators (diaryliodonium salt) and co-initiator can be calculated from the classical Equation (1),

ΔG_et_ = F[E_ox_ (D/D^•+^) − E_red_ (A^•−^/A)] − E^00^ − (Ze^2^/εa) (1)

where F is the Faraday constant (F = 96485.33289(59) C mol^−1^), E_ox_ (D/D^•+^), E_red_ (A^•−^/A), E_00_ and (Ze^2^/εa) are the oxidation potential of the co-initiator, the reduction potential of the diaryliodonium salt, the excited state energy and the electrostatic interaction energy for the initially formed ion pair, respectively. Parameter (Ze^2^/εa) is generally considered negligible in polar solvents.

### 2.4. Preparation of Thin-Layer Samples for Monitoring the Photopolymerization Processes by FPT

The compositions for FPT measurements were prepared by dissolution of the photoinitiator and each fluorescent sensor in the monomer in such proportions as to obtain the concentration 1.0% by weight of the photoinitiator and 3.69 × 10^−3^ [mol/dm^3^] of the sensor. Before measurement, two drops of the composition were placed in the middle of a microscope slide (75 mm × 25 mm × 1 mm, from Thermo Scientific), equipped with two 0.09 mm thick spacers located on the slide sides, and, the slide was covered with another microscope slide to form a sandwich structure. The slides were kept together using paper clips placed on their sides. Thickness of the samples was measured with an electronic micrometer.

### 2.5. Monitoring the Photopolymerization Processes by FPT

The cure monitoring system was composed of a microcomputer-controlled miniature spectrometer (SilverNova from StellarNet, Inc., Tampa, FL, USA), a sensor head where the thin-layer sample was placed during measurement, and a UV LED emitting at 320 nm (UVTOP315-BL-TO39, from Roithner LaserTechnik GmbH, Wien, Austria), equipped with an appropriate power supply. The sensor head was the same as the one described previously [37]; however, it has been modernized with a thermostat system that guarantees the stability of environmental conditions during the monitoring of the photopolymerization process. Therefore, all photopolymerization processes were carried out at an ambient temperature (25 °C) using ITC4020 thermostat (from Thorlabs Inc., Newton, NJ, USA). The UV light from the LED illuminated about 5 mm spot within the thin-layer sample. The light from the measurement site was transferred to the spectrometer using a PMMA fiber optic cable with 2 mm core. The UV LED was supplied with constant current of 23 mA from an appropriate stabilized constant current source.

### 2.6. Monitoring the Photopolymerization Processes by Real-Time FT-IR

#### 2.6.1. Cationic Photopolymerization (CP) Experiments

The photocurable formulations (25 μm thick) were deposited on a BaF_2_ pellet under air. The evolution of the epoxy group content was continuously followed by real-time FT-IR spectroscopy (Nicolet iS10, from Thermo Scientific, Waltham, MA, USA) at about 790 cm^−1^. For vinyl monomer (TEGDVE), the photopolymerization was followed at about 1640 cm^−1^.

#### 2.6.2. Free-Radical Photopolymerization (FRP) Experiments

The experiments were carried out in laminated conditions. The PP films (25 μm thick) deposited on a horizontal holder for FT-IR spectrometer were irradiated. The evolution of the double bond of acrylate TMPTA content was continuously followed by real time FT-IR spectroscopy (Nicolet iS10, from Thermo Scientific, Waltham, MA, USA) at about 1634 cm^−1^.

#### 2.6.3. Thiol-ene Photopolymerization (FRP) Experiments

The photosensitive formulations were deposited on a BaF_2_ pellet. The evolution of the thiol (S-H) group content was continuously followed by real time FT-IR spectroscopy (Nicolet iS10, from Thermo Scientific, Waltham, MA, USA) at about 2570 cm^−1^. FT-IR also followed the double bond conversion at about 1637 cm^−1^. A stoichiometric ratio of thiol vs. ene was used in all the experiments.

#### 2.6.4. Source of Light for Real-Time Experiments

The light source for the real-time FT-IR method was the 365 nm M365L2c UV-LED diode (from Thorlabs Inc., Tampa, FL, USA powered by a DC2200 regulated power supply (from Thorlabs Inc., Tampa, FL, USA). The UV-LED diode was started 10 s after the start of spectral registration.

## 3. Results

### 3.1. Spectral Characteristics of the Sensors/Co-Initators

**Figure 1:**
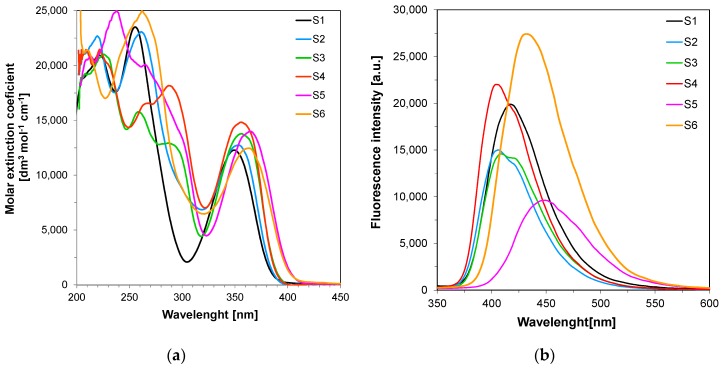
Spectroscopic properties of 2-amino-4,6-diphenyl-pyridine-3-carbonitrile derivatives: (**a**) UV-visible absorption spectra of the 2-amino-4,6-diphenyl-pyridine-3-carbonitrile derivatives in acetonitrile; (**b**) Fluorescence spectra of the 2-amino-4,6-diphenyl-pyridine-3-carbonitrile derivatives in acetonitrile in extinction 320 nm and integration time 1 s.

The ground state absorption spectra of the proposed 2-amino-4,6-diphenyl-pyridine-3-carbonitrile derivatives are depicted in Figure 1. All 2-amino-4,6-diphenyl-pyridine-3-carbonitrile derivatives absorb strongly at near UV range of spectrum, with the long-wavelength absorption maximum within the range 349–364 nm. The extinction coefficient at the peak maximum of the absorption spectrum was usually within 10,000–15,000 [dm^3^·mol^−1^·cm^−1^], which is high enough for application of the 2-amino-4,6-diphenyl-pyridine-3-carbonitrile derivatives as fluorescent sensors for polymer application. The investigated compounds exhibit fluorescence in blue light region with intensity maximum at around 400–450 nm (Table 2), which also makes these compounds good candidates for the role of fluorescent sensors.

The effects of substituents on absorption and fluorescence of the 2-amino-4,6-diphenyl-pyridine-3-carbonitrile derivatives are clearly visible, as significant differences in absorption and fluorescent spectra were observed for different derivatives of S1. These effects were dictated by structural changes in the basic chromophore system 2-amino-4,6-diphenyl-pyridine-3-carbonitrile (S1). The introduction of additional electron donor groups (e.g., methoxy group) to the compound S1 as a basic system and the electron acceptor group (e.g., nitrile group) resulted in shifting the absorption and fluorescence characteristics for S5 and S6 towards longer wavelengths. The observed bathochromic effect results from the fact that S5 and S6 is an interesting system of organic -systems, end-capped with an electron donor (D) and an electron acceptor (A) that represent molecules widely known as push-pull systems.

### 3.2. Performance of the 2-amino-4,6-diphenyl-pyridine-3-carbonitrile derivatives in the Role of Sensors in FPT

**Figure 2:**
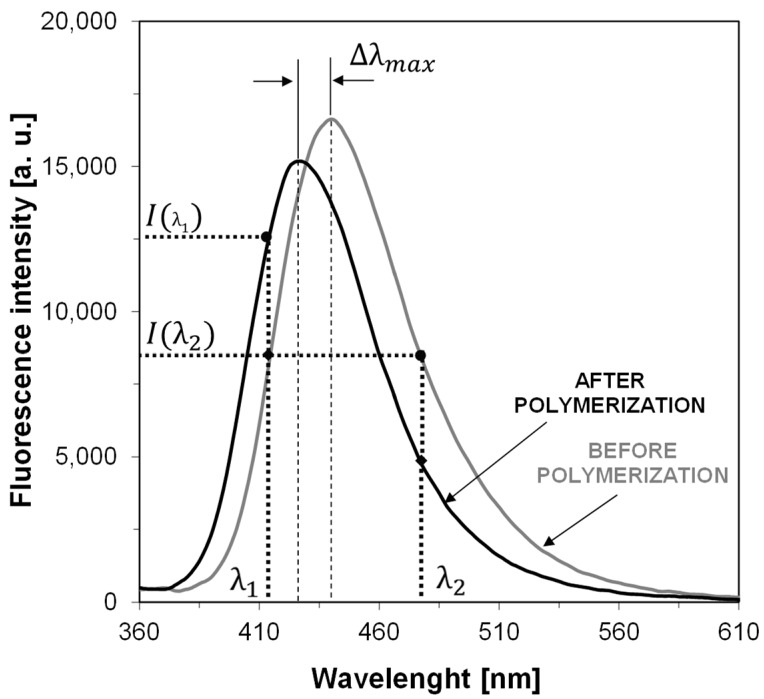
Changes of fluorescence spectra of example 2-amino-4-(4-cyanophenyl)-6-(4-methoxyphenyl) pyridine-3-carbonitrile sensor (S5) during free radical photopolymerization of TMPTMA monomer under irradiation 320 nm, (λ_1_, λ_2_ are monitoring wavelengths).

**Figure 3:**
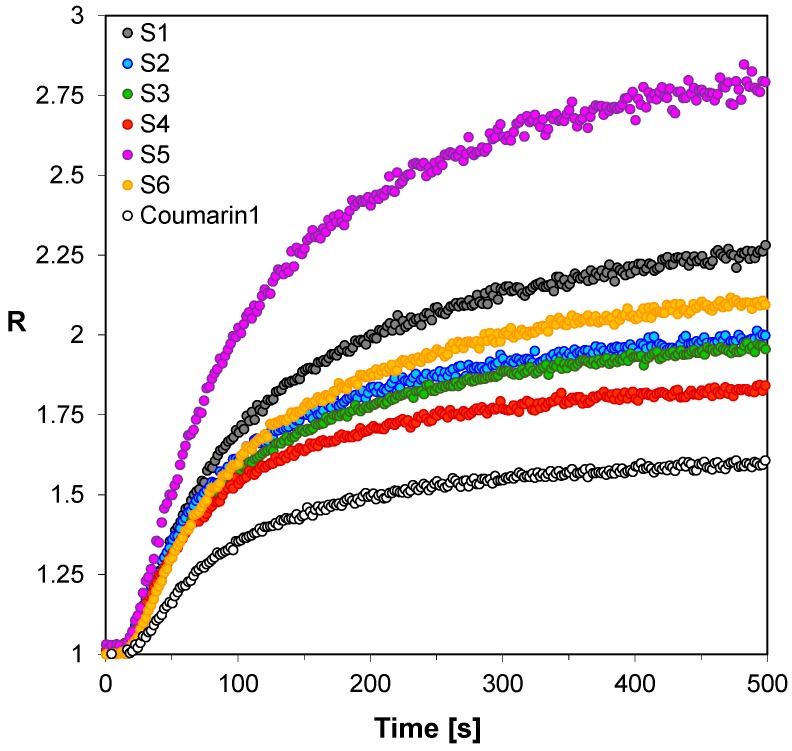
Monitoring free-radical photopolymerization of TMPTMA monomer under 320 nm by FPT, using the 2-amino-4,6-diphenyl-pyridine-3-carbonitrile derivatives (S1–S6) as the fluorescent sensors and Coumarin 1 (C1) as a reference probe.

First, in order to verify whether the 2-amino-4,6-diphenyl-pyridine-3-carbonitrile sensors (S1–S6) would be applicable for monitoring of free-radical photopolymerization of acrylic monomers, their fluorescence spectra were recorded in a photocurable composition based on TMPTMA monomer during continuous irradiation of the sample with UV light at 320 nm until the polymerization was complete. Figure 2 shows example fluorescence spectra of the sensor S5 recorded at equal time periods during the polymerization process. The changes of fluorescence characteristics of the other 2-amino-4,6-diphenyl-pyridine-3-carbonitrile derivatives were similar to those of the sensor S5. All the tested sensors (S1–S6) shifts towards shorter wavelengths during free-radical photopolymerization. Such a shift by several nanometers is a typical behavior of most fluorescent sensors, which is the result of changes in polarity and microviscosity of the medium surrounding the sensor molecules. This behavior follows directly from the increase of the medium rigidity around the sensor molecules, and reduced polarity during the polymerization process. The conversion of unsaturated bonds in the monomer into saturated ones within the polymer structure results in decrease of the polarity of the system. To monitor the on-line and in-situ changes during the photopolymerization process, the parameter used was the ratio of the fluorescence intensity ratio (R) measured at two different wavelengths λ_1_ and λ_2_, which were located on both sides of the maximum fluorescence spectrum of the probe at half its height. In this approach, the fluorescence intensity ratio (R) was used as a quantitative indicator of the polymerization progress (Figure 2). The monitoring wavelengths (λ_1_ and λ_2_) were selected individually for each probe to correspond to half of the fluorescence intensity at the peak maximum before polymerization. In this way, the ratio always started from 1 and increased with the polymerization progress. Figure 3 shows the photopolymerization profiles obtained in terms of the ratio (R) using the sensors studied. All sensors (S1–S6 and C1) shifted their spectrum sufficiently to enable precise monitoring of the photopolymerization progress using the fluorescence intensity ratios as the progress indicator. The sensitivity of the tested compounds was also determined during the free-radical photopolymerization process. Sensitivity of the 2-amino-4,6-diphenyl-pyridine-3-carbonitrile derivatives, defined as the ratio span ((R_max_ − R_0_)/R_0_) between an uncured and cured state of the composition, depends on the type of substituents. This sensitivity is even twice higher to the sensitivity of Coumarin 1 used as a reference. The 2-amino-4-(4-cyanophenyl)-6-(4-methoxyphenyl)-pyridine-3-carbonitrile (S5) showed the highest sensitivity within the series of the pyridine derivatives studied (Table 3).

This can be explained when the sensor sensitivity being proportional to the magnitude of push-pull effect of substituents on the fluorescing molecule is considered. Slightly lower sensitivity is displayed by a 2-amino-6-(4-cyanophenyl)-4-(4-methoxyphenyl)pyridine-3-carbonitrile (S6), which is a derivative that shows the reverse position of the methoxy and nitrile substituents relative to the sensor S5. The reduced sensitivity for the S6 sensor can be explained by the presence of an electron-withdrawing substituent (e.g., -cyanophenyl as it is in S6) in close proximity to the pyridine ring. This is due to the fact that it is more beneficial, from the point of view of the researched sensors’ application, to place an electron-donor substitution phenyl at the sixth position of the 2-amino-4,6-diphenyl-pyridine-3-carbonitrile chromophore, as is the case of the S5 sensor. This is most likely to be linked to the fact that the pyridine ring itself decreases the p-electron density on the carbon atoms and are thus p-deficient in comparison to benzene. It is noteworthy, that in the case of derivatives which did not contain electron-acceptor groups in the 2-amino-4,6-diphenyl-pyridine-3-carbonitrile chromophore, only the electron-donating groups show a lower sensitivity of 50% compared to the S5 sensor. For the following sensors: S2, S3 and S4, regardless of the location of the methoxy moiety in the chromophore, the sensitivity levels are similar. This proves that the introduction of an additional group with an opposite nature increases the sensitivity of the probe, additionally in the 2-amino-4,6-diphenyl-pyridine-3-carbonitrile the place of substitution of these groups has a very high importance, which was shown on the examples of S5 and S6. All spectroscopic data obtained during the monitoring of this process is presented in the table.

In industrial practice, the free-radical photopolymerization systems are most commonly used. The basis of widely used free-radical photopolymerization systems have been methacrylate monomer, which polymerize in accordance with the radical mechanism. The reason for their popularity is their high reactivity and possibility to obtain materials of varied properties, which result from the possibility of making numerous modifications of ester chain. For this reason, it is so important to strictly monitor the polymerization progress that would be applicable directly in production. The above-described derivatives of 2-amino-4,6-diphenyl-pyridine-3-carbonitrile meet all the requirements for the fluorescent sensors to be utilized in the monitoring of free-radical photopolymerization processes by FPT method.

**Figure 4:**
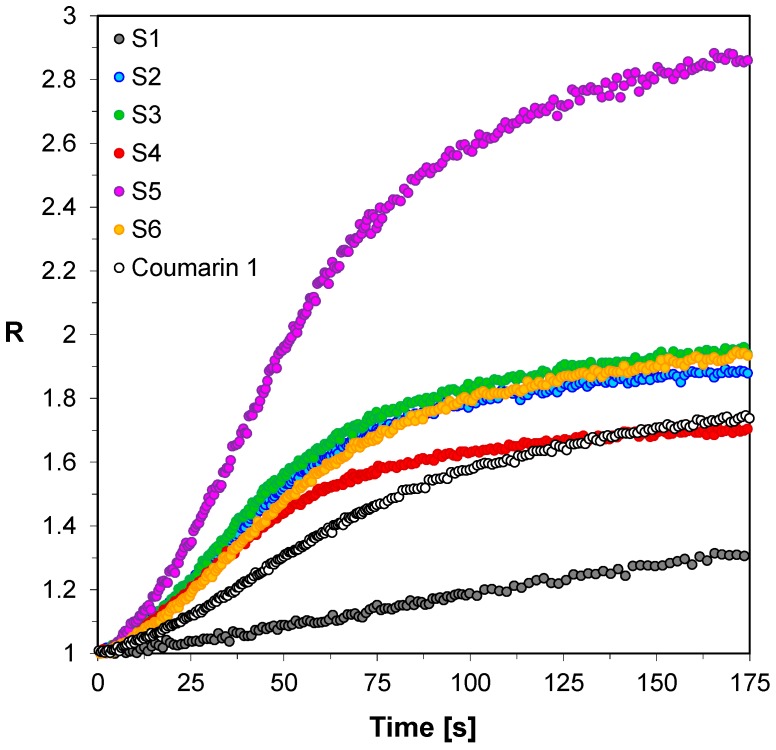
Monitoring thiol-ene photopolymerization of TMPTMA/MERCAPTO (50%/50% *w*/*w*) monomer under 320 nm by FPT, using the 2-amino-4,6-diphenyl-pyridine-3-carbonitrile derivatives (S1-6) as the fluorescent sensors and Coumarin 1 (C1) as a reference probe.

Another important type of photopolymerization process from the industrial point of view is thiol-ene photopolymerization. The popularity of this type of photopolymerization results from its similarity to the process of free-radical photopolymerization but shows the resistance to the inhibiting action of atmospheric oxygen. Thiol-ene photopolymerization is the process of polyaddition based on the stoichiometric reaction of multi-functional alkenes (“enes”) with thiols. The resistance to atmospheric oxygen results from the fact that thiols are effective carriers of the chain and the reaction with oxygen progresses very rapidly with the simultaneous regeneration of the propagating species, i.e., thiyl radical. Therefore, thiol-ene polymerization has become an attractive photopolymerization method not only because of resistance of oxygen inhibition, but also because of its many salient features such as low stress build-up, and narrow glass transition temperatures. These features are a direct result of the mechanism through which the network is formed. Accordingly, the usefulness of 2-amino-4,6-diphenyl-pyridine-3-carbonitrile derivatives for the role in monitoring of thiol-ene photopolymerization was also examined. Based on the shape of the kinetic curves obtained by the FPT method (Figure 4), it can be concluded that all tested 2-amino-4,6-diphenyl-pyridine-3-carbonitrile derivatives allow monitoring the changes occurring during thiol-ene photopolymerization using the fluorescence intensity ratio (R). For all sensors (S1–S6) tested, it can be seen that they react to changes from the very beginning of the process, which is manifested by the increase in R and the steep inclination of the R dependence on time. As the photopolymerization process progresses, the photopolymerization rate gradually decreases and eventually disappears, resulting in a plateau on the kinetic curves (Figure 4). The tested sensors S1–S6 differ in their sensitivity, which is indicated by the different range of variability of the parameter R. All spectroscopic data from kinetic studies from thiol-ene photopolymerization process are presented in Table 3. From among the sensors studied during thiol-ene photopolymerization, the sensor S5 also turned out to be the most sensitive to changes occurring in its environment during the monomers’ photopolymerization, when R was used as the indicator (Table 3). The sensitivity of sensor S5 is the highest; it is about three times higher than that of Coumarin 1 used as a reference. The increase of the ratio (R) with progress of monomers’ polymerization comes from the shift of the fluorescence spectrum to shorter wavelengths. The shift of the fluorescence spectrum to a shorter wavelength during the monomer polymerization indicates that the system polarity decreased, which is typical for unsaturated monomers, because during the polymerization more polar double bonds of monomers are converted into less polar single bonds in the polymer. In the case of sensor S5 this shift was 14 nm, which can be considered as being optimal for cure monitoring using the ratio (R). A larger shift would cause passing the fluorescence spectrum over its maximum at the wavelength λ_1_, which would lead to a significant decrease of the probe sensitivity at high degrees of cure, because then both monitoring wavelengths would switch to the same side of the spectrum. In conclusion, it was clearly demonstrated that all 2-amino-4,6-diphenyl-pyridine-3-carbonitrile derivatives (S1-6) enable monitoring of the thiol-ene photopolymerization progress using the fluorescence intensity ratio (R) as the progress indicator.

**Figure 5:**
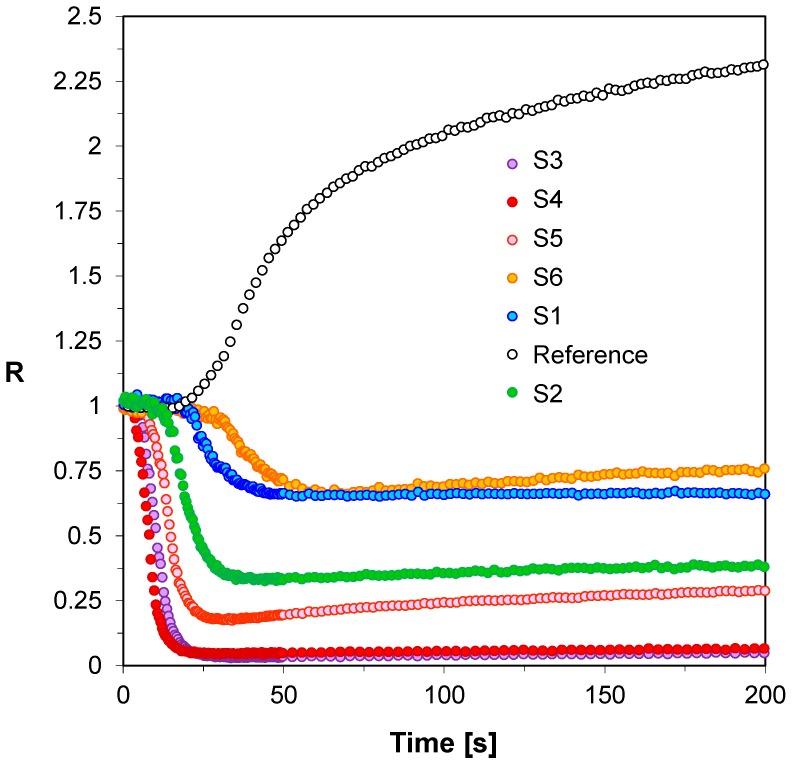
Monitoring cationic photopolymerization of TEGDVE monomer under 320 nm by FPT, using the 2-amino-4,6-diphenyl-pyridine-3-carbonitrile derivatives (S1–S6) as the fluorescent sensors and 25ST as a reference probe.

**Figure 6:**
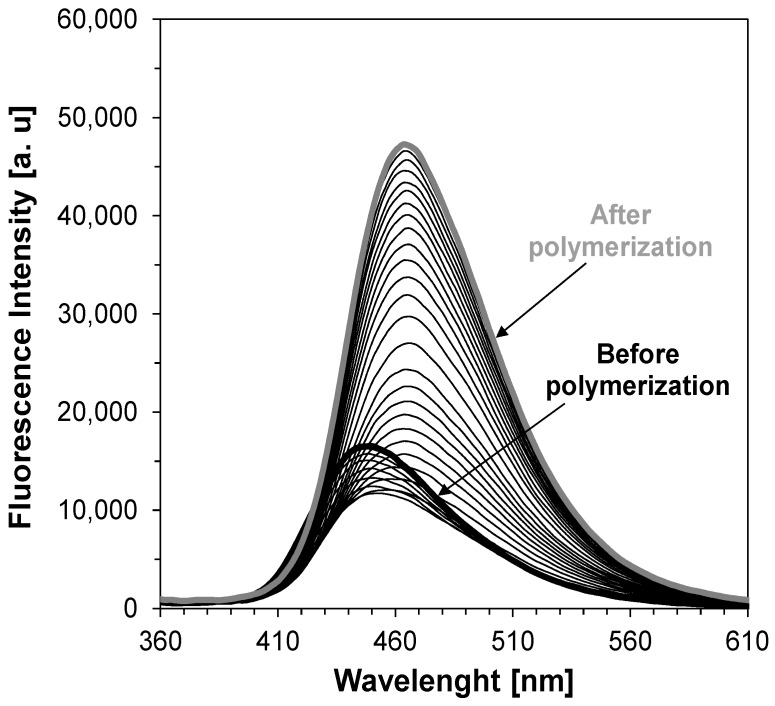
Changes of fluorescence spectra of 2-amino-4-(4-cyanophenyl)-6-(4-methoxyphenyl)pyridine-3-carbonitrile sensor (S5) of cationic photopolymerization of TEGDVE monomer under irradiation 320 nm.

Encouraged by these positive results for free-radical and thiol-ene photopolymerization processes, it was decided to examine the usefulness of the developed 2-amino-4,6-diphenyl-pyridine-3-carbonitrile derivatives (S1–S6) for monitoring cationic photopolymerization process of vinyl monomer (TEGDVE). Initially, for monitoring the progress of cationic photopolymerization of TEGDVE monomer with the 2-amino-4,6-diphenyl-pyridine-3-carbonitrile derivatives (S1–S6) as a fluorescent sensor, the fluorescence intensity ratio (R), (i.e., in the same way as that used for free-radical and thiol-ene photopolymerization processes), was applied (Figure 5). A striking difference in the behavior of the sensors (S1–S6) in the TEGDVE monomer studied is that R decreased with the progress of photopolymerization of TEGDVE, which is the opposite behavior to in the case of the free-radical and thiol-ene photopolymerization of monomers. The specific behavior of sensors (S1–S6) during this type of polymerization results from the fact that during the cationic polymerization process, a strong proton acid is generated under photolysis of iodonium salt. But in this situation, in the first step the strong protic acid (hexafluorophosphoric acid) is consumed for protonation of the fluorescent sensors (S1–S6), because these compounds contain basic amino groups in their structures as is clearly indicated by the drastic decrease in the fluorescence intensity of the sensor at the first seconds of cationic polymerization (Figure 6).

**Figure 7:**
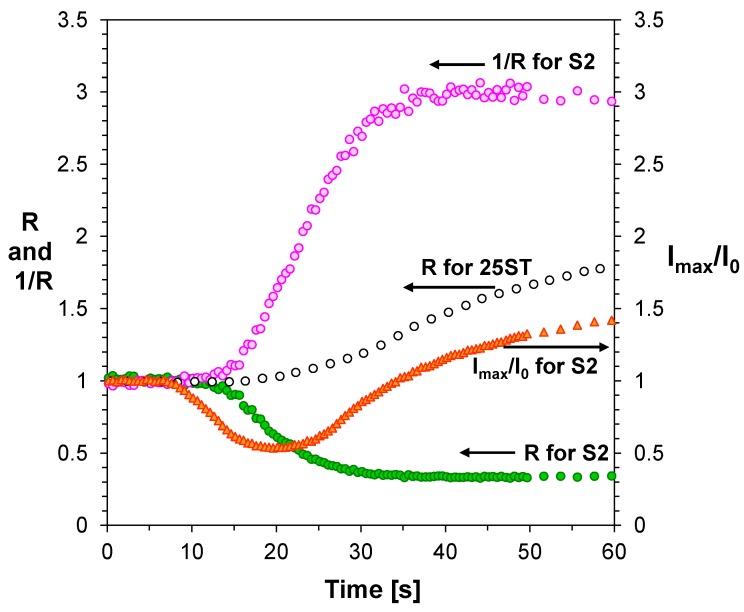
Kinetic profiles of cationic photopolymerization of TEGDVE monomer, obtained by FPT method, using 2-amino-4-(4-methoxyphenyl)-6-phenyl-pyridine-3-carbonitrile sensor (S2) and different kinetic parameters.

In each case, after the induction period, where fluorescent sensors were protonated, and a sufficient amount of hexafluorophosphoric acid was generated from the photoinitiator, monitoring of the cationic polymerization began, as indicated by a sharp increase of the intensity of fluorescent spectrum of sensors. As confirmation of this explanation, the changes of the fluorescence spectrum of the S2 during the cationic photopolymerization process can be indicated (Figure 6). Moreover, it was confirmed that the fluorescence intensity ratio (R), is not a reliable indicator of the polymerization progress in the case of vinyl ethers, such as TEGDVE. To find the answer to the question of what kind of parameter should be used for monitoring changes occurring during cationic photopolymerization of TEGDVE monomer, the response of sensors S1–S6 was compared to the response of 25ST probe, which was previously found to be a good probe for monitoring cationic photopolymerization processes using fluorescence intensity ratio [47]. For this purpose, the same TEGDVE monomer, the same concentration of HIP photoinitiator and identical measurements conditions was prepared for composition with S2 and composition with 25ST as a reference. The results are shown in Figure 7.

Nevertheless, when we wanted to use the 2-amino-4,6-diphenyl-pyridine-3-carbonitrile derivatives as fluorescent sensors for cationic photopolymerization, the reciprocal of R and the normalized fluorescence intensity (I_max_/I_0_) were used. The parameter (1/R) corresponds to the ratio of fluorescence intensity at a longer wavelength to that at a shorter wavelength, in turn the normalized fluorescence intensity (I_max_/I_0_) is the intensity (I_max_) measured at the particular irradiation times, which is normalized to the initial intensity (I_0_) before polymerization. When we compared the received kinetic profiles for tS5 and 25ST with R parameters (to be exact: parameters R for sensors S5 and 25ST and parameter 1/R for sensors S5), it was clear that the photopolymerization conditions were identical, because the induction times for these kinetic profiles are identical and were around 15 s (Figure 7). On the other hand, it is clearly seen that monitoring of cationic photopolymerization processes using the normalized fluorescence intensity (I_max_/I_0_) only enables monitoring the protonation process of 2-amino-4,6-diphenyl-pyridine-3-carbonitrile derivatives during the cationic polymerization. Therefore, only the course of the polymerization process when it is used for monitoring the parameter (1/R) indicates that sensor S2 not only monitors the polymerization progress, but also accelerates the cationic photopolymerization, probably by electron transfer from some of the excited S2 molecules to HIP photoinitiator; which speeds up generation of initiating species from HIP. Moreover, a slight decrease of the (1/R) of the S2 band at high monomer conversions before reaching the plateau indicates that compound S2 is consumed under the cationic photopolymerization process.

### 3.3. Performance of the 2-amino-4,6-diphenyl-pyridine-3-carbonitrile derivatives in the Role of Co-Initiators in Bimolecular Photoinitiating Systems for Cationic Photopolymerization

During our studies of the performance of 2-amino-4,6-diphenyl-pyridine-3-carbonitrile derivatives in the role of sensors, we noticed that the cationic photopolymerization did not occur when the probe was not added to the composition; the monomer remained liquid even after extended irradiation time at 365 nm wavelength. The lack of cationic polymerization at this wavelength results from the fact that the diphenyliodonium hexafluorophosphate does not absorb at that wavelength. However, it was surprising that the photopolymerization occurred rapidly in the presence of both the photoinitiator and a 2-amino-4,6-diphenyl-pyridine-3-carbonitrile derivatives upon 320 nm and also upon 365 nm. This indicated that these sensors participated in the cationic photopolymerization process and what is more, it was necessary for polymerization to occur. To evaluate the effect of the 2-amino-4,6-diphenyl-pyridine-3-carbonitrile derivatives on cationic photopolymerization of vinyl (TEGDVE) and cycloaliphatic epoxide (CADE) initiated with diphenyliodonium hexafluorophosphate, a series of experiments were performed using real time FT-IR and electrochemical analysis. The results obtained for these derivatives are shown in Table 3. The long-wavelength absorption characteristics of the 2-amino-4,6-diphenyl-pyridine-3-carbonitrile derivatives almost perfectly matched the emission intensity of near UV-LED with the maximum of emission at 365 nm, which are environmentally friendly sources of light currently used in the photochemistry industry (Table 4).

**Figure 8:**
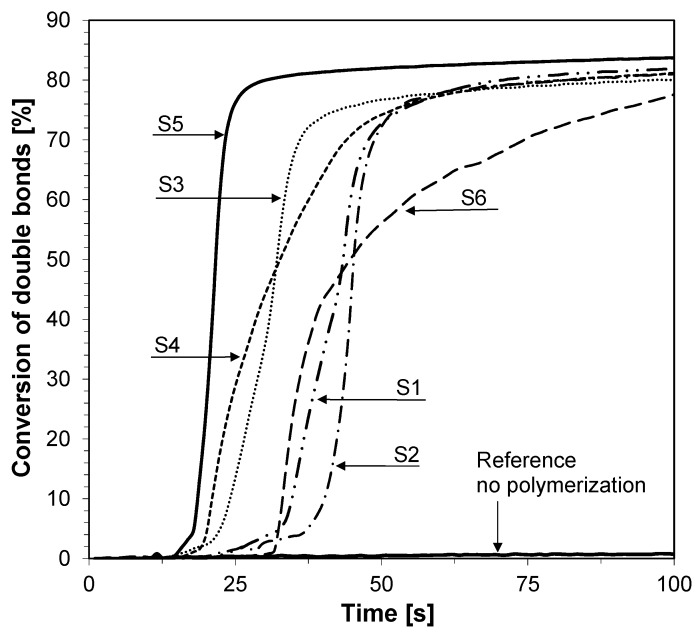
Polymerization profiles of TEGDVE (vinyl function conversion vs irradiation time) upon exposure to the LED@365 nm (95 mW/cm^2^) under laminate in the presence of different photoinitiating systems based on diphenyliodonium hexafluorophosphate (HIP, 1%) and 2-amino-4,6-diphenyl-pyridine-3-carbonitrile derivatives. The irradiation starts at t = 10 s.

The photopolymerization of vinyl monomer (TEGDVE) was carried out in laminate using the UV-LED 365 nm (95 mW/cm^2^). The diphenyliodonium hexafluorophosphate alone does not lead to any polymerization (Figure 8); nevertheless, in the presence of the 2-amino-4,6-diphenyl-pyridine-3-carbonitrile derivatives that are only at 0.1% in the bimolecular photoinitiating system based on iodonium salt (1% by weight), an excellent polymerization is noted with a conversion level of about 85% after 50 s of irradiation.

**Figure 9:**
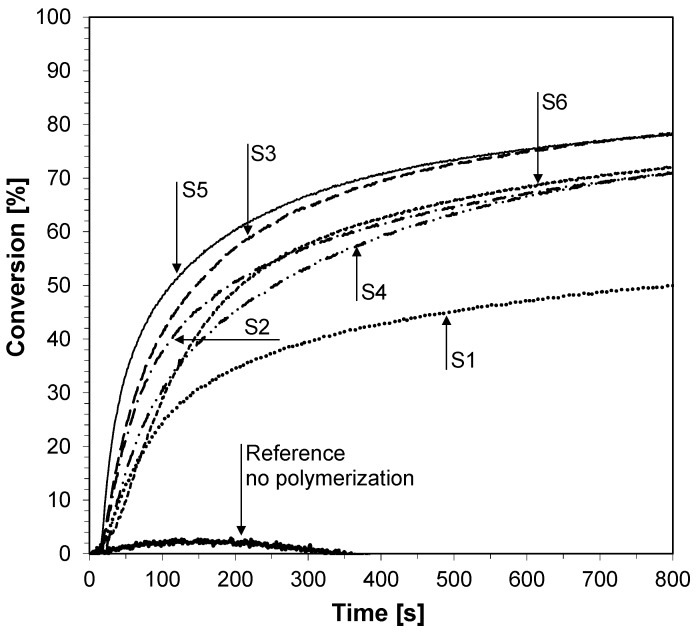
Polymerization profiles of CADE (epoxy function conversion vs. irradiation time) upon exposure to the LED@365 nm (190 mW/cm^2^) under air in the presence of different photoinitiating systems based on diphenyliodonium hexafluorophosphate (HIP, 1%) and 2-amino-4,6-diphenyl-pyridine-3-carbonitrile derivatives. The irradiation starts at t = 10 s.

**Figure 10:**
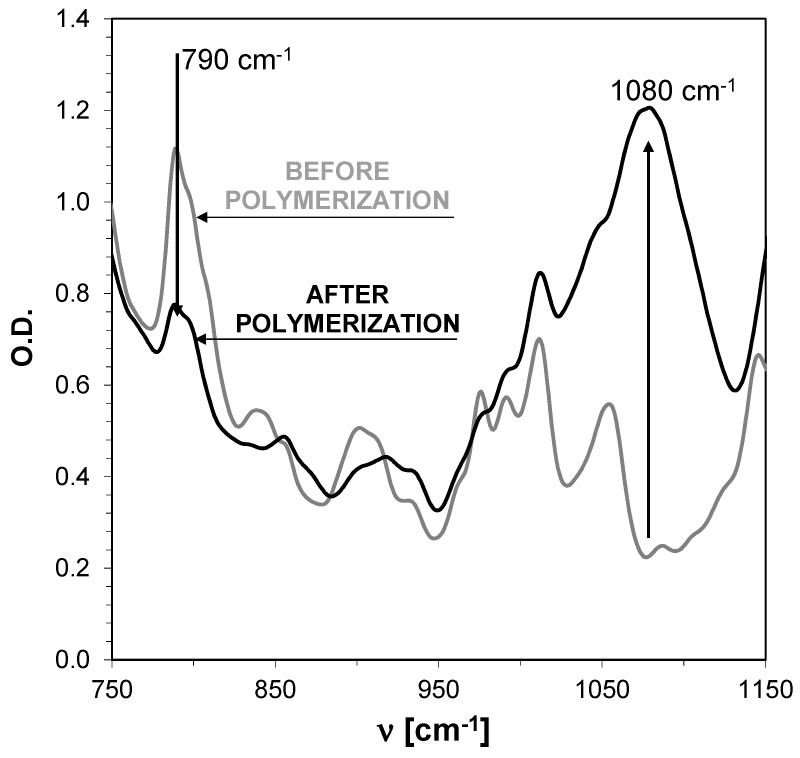
FTIR spectra of C-H bonds band in oxirane ring before and after photopolymerization of CADE under UV-LED 365 nm (190 mW/cm^2^) irradiation in composition with S5.

The photoinduced ring opening cationic photopolymerization of cycloaliphatic epoxide monomer (CADE) in the presence of two-component photoinitiating systems based on 2-amino-4,6-diphenyl-pyridine-3-carbonitrile derivatives and diphenyliodonium hexafluorophosphate (HIP) in combinations (0.1%/1% *w*/*w*) under air was also investigated under UV-LED exposure at 365 nm. Typical epoxy conversion versus time profiles is illustrated in Figure 9 while the final conversion of light irradiation is summarized in Table 4 along with the diphenyliodonium hexafluorophosphate used as the reference. All these photoinitiating systems exhibit a very high efficiency in terms of final epoxy function conversion of nearly 80% (Figure 9 and Table 4). A new peak ascribed to the polyether network arises at 1080 cm^−1^ (Figure 10) in the FT-IR spectra. As mentioned below, the diphenyliodonium hexafluorophosphate alone does not activate the polymerization (functional group conversion is 0% using HIP alone), indicating the role of 2-amino-4,6-diphenyl-pyridine-3-carbonitrile derivatives as the co-initiator of the iodonium salt decomposition upon near-UV to visible light LEDs. It is worth noting that the difference between the vinyl monomer versus cycloaliphatic epoxy monomer profiles should be ascribed to the faster polymerization reactions usually observed with vinyl ethers. Nevertheless, when we compared the kinetic profile of photopolymerization of epoxy monomer (CADE), it is shown that the S1/HIP is the slowest photoinitiating system out of all investigated systems. This is mainly associated with the characteristic of absorption of the S1 (Table 4), because sensors (S1) have the lowest molar extinction coefficients compared to others 2-amino-4,6-diphenyl-pyridine-3-carbonitrile at 365 nm.

The second factor influencing the effectiveness of photoinitiation for the S1/HIP system is the electrochemical properties of the 2-amino-4,6-diphenyl-pyridine-3-carbonitrile co-initiator (S1) (Table 4), which is related to free energy change ΔG_et_ for an electron transfer reaction between the fluorescent sensor as the electron donor and HIP as an electron acceptor. The electron transfer from the excited co-initiators to iodonium salt (HIP) is feasible if the change in free energy (ΔG_et_) is negative. For this reason, the free energy change ΔG_et_ can be calculated according to the classical equation number 1. To demonstrate the feasibility of an electron transfer process between fluorescent sensors and HIP, cyclic voltammograms (CV) of the sensors were also determined. The electrochemically determined potentials of the oxidation of half-wave of the studied sensors and the calculated ΔG_et_ values are listed in Table 3. As can be seen, the ΔG_et_ values are all negative and electron transfers from all excited 2-amino-4,6-diphenyl-pyridine-3-carbonitrile derivatives to iodonium salt are thermodynamically favorable. However, it seems that depending on the oxidative potential of the 2-amino-4,6-diphenyl-pyridine-3-carbonitrile, the value of the calculated ΔG_et_ is different. The sensor/co-initiator S1 exhibit the highest oxidative potential and thus the highest value of ΔG_et_. For this reason, the efficiency of the photoinitiating system based on S1/HIP is at the worst level during cationic photopolymerization of CADE.

**Figure 11:**
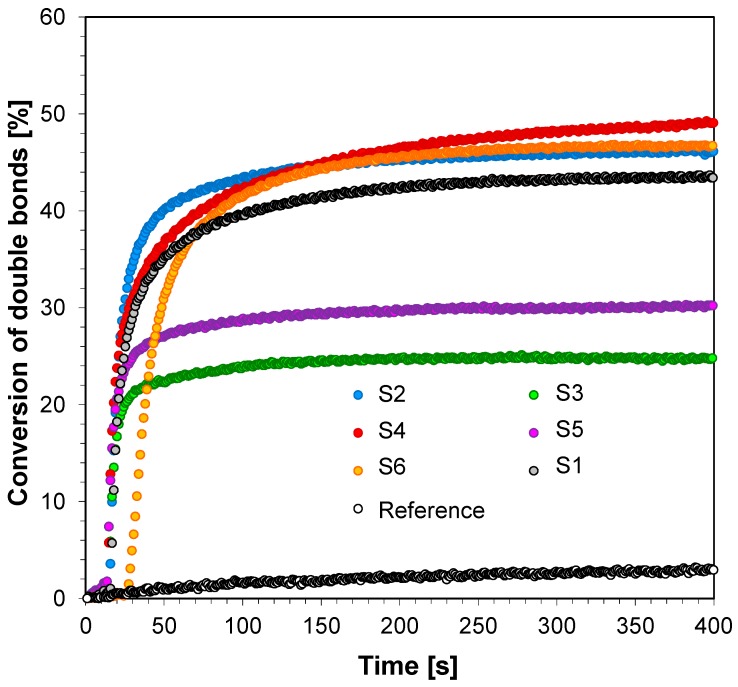
Conversion of double bonds of TMPTA recorded during free-radical photopolymerization with binary initiating system—HIP and fluorescent probes under UV-LED 365 nm (95 mW/cm^2^) irradiation.

The ability of the bimolecular photoinitiating systems based on the 2-amino-4,6-diphenyl-pyridine-3-carbonitrile derivatives and iodonium salt to initiate the free radical promoted photopolymerization of trimethylolpropane triacrylate (TMPTA) in laminate was also investigated. Conversion time profiles for the free radical photopolymerization of TMPTA in laminate upon exposure to UV-LED is shown in Figure 11 and the final methacrylate function conversions are listed in Table 5. The radical polymerization of TMPTA in laminate using the 2-amino-4,6-diphenyl-pyridine-3-carbonitrile derivatives and iodonium salt combinations is also feasible under soft ultraviolet light radiation. The efficiencies of the free radical photopolymerization of TMPTA can be easily increased by increasing the power of the used LED light sources.

**Figure 12:**
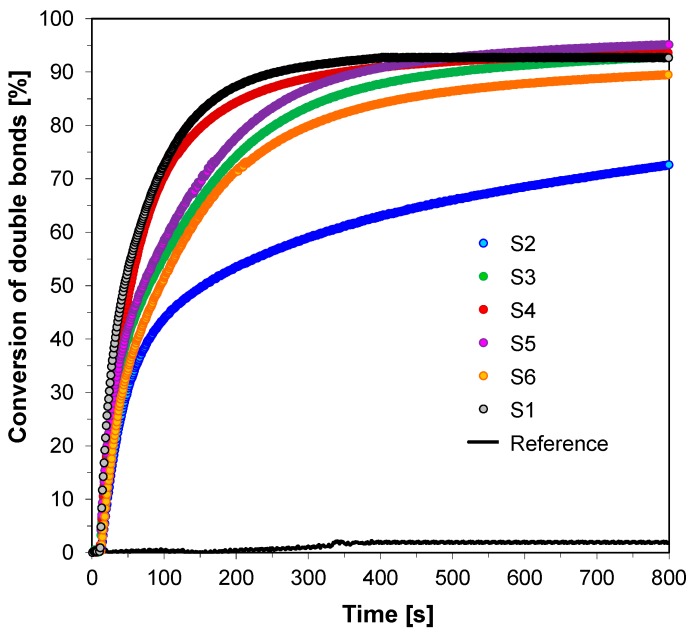
Conversion of double bonds of TMPTMA recorded during thiol-ene photopolymerization of TMPTMA and MERCAPTO composition with binary initiating system—HIP and fluorescent probes—under UV-LED 365 nm (95 mW/cm^2^) irradiation.

**Figure 13:**
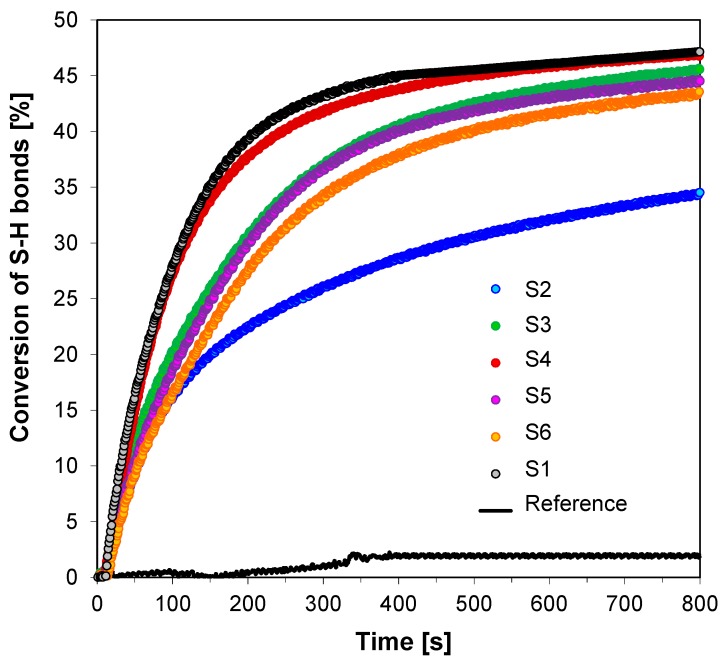
Conversion of S-H bonds of MERCAPTO recorded during thiol-ene photopolymerization of TMPTMA and MERCAPTO composition with binary initiating system—HIP and fluorescent probes—under UV-LED 365 nm (95mW/cm^2^) irradiation.

Excellent polymerization profiles were also obtained for thiol-ene photopolymerization process of trimethylolpropane trimethacrylate (TMPTMA) and trimethylolpropane tris(3-mercaptopropionate) (MERCAPTO). The vinyl double bond conversions of TMPTMA are higher (73% and 95%) (Figure 12) than those of thiol (35% and 46%) (Figure 13), which could be attributed to the fact that TMPTMA can also be efficiently homopolymerized by the radicals generated from the 2-amino-4,6-diphenyl-pyridine-3-carbonitrile derivatives/iodonium salt (HIP) combination throughout the thiol−ene photopolymerization process. The lower final conversion of the trithiol indicates that the light induced free radical homopolymerization of methacrylate monomer probably predominates and the thiol−ene process occurs to a lesser extent.

Consequently, the 2-amino-4,6-diphenyl-pyridine-3-carbonitrile derivatives can be used in a dual role: (a) as fluorescent sensors for monitoring the cationic polymerization progress using the reciprocal fluorescence intensity ratio (1/R), and (b) as co-initiators for long-wavelength photoinitiating system for cationic, free-radical and thiol-ene photopolymerization with diphenyliodonium photoinitiators.

## 4. Conclusions

All 2-amino-4,6-diphenyl-pyridine-3-carbonitrile derivatives (S1-6) shift their fluorescence spectrum to shorter wavelengths upon free-radical and thiol-ene photopolymerization of the medium, which enables observation of the polymerization progress using the fluorescence intensity ratio (R) as the progress indicator. Hence, these probes can be applied for monitoring free-radical and thiol-ene polymerization processes using the FPT method both off-line and online within a broad range of monomer conversions. Depending on the type of substituent in the 2-amino-4,6-diphenyl-pyridine-3-carbonitrile chromophore, the sensors exhibit different sensitivity to changes occurring in their microenvironment. The sensor S5 is the most sensitive.

The response of the 2-amino-4,6-diphenyl-pyridine-3-carbonitrile derivatives (S1-6) in cationic polymerization of TEGDVE monomer is completely different and is not synonymous to the response registered during the monitoring of the free-radical or thiol-ene polymerization processes. The 2-amino-4,6-diphenyl-pyridine-3-carbonitrile derivatives (S1-6) act as long-wavelength photosensitizers for cationic photopolymerization of TEGDVE and CADE monomers initiated with diphenyliodonium hexafluorophosphate photoinitiator. The accelerating effect of the probe on the polymerization rate compensates in excess the effect of slight consumption of the hexafluorophosphoric acid in the first stage of cationic photopolymerization. Moreover, the addition of the probe results in the possibility of photopolymerization under 365 nm wavelength of UV light. Finally, it is worth emphasizing that the diphenyliodonium photoinitiators alone do not work at that wavelength. The 2-amino-4,6-diphenyl-pyridine-3-carbonitrile derivatives (S1-6) in combination with an iodonium salt (HIP) can be also used as high performance UV-A light sensitive bimolecular photoiniationg systems to efficiently initiate the free radical and thiol−ene photopolymerization processes as well. For these reasons, this research extends the range of high-performance photoinitiating systems operating under UV-LED sources with maximum emissions at 365 nm.

In conclusion the 2-amino-4,6-diphenyl-pyridine-3-carbonitrile derivatives (S1-6) described in this paper can be applied simultaneously in double roles: as fluorescent probes for cure monitoring of photocurable compositions by the FPT method, and as a photosensitizer for iodonium photoinitiators to initiate the different photopolymerization processes under near UV light.

## Figures and Tables

**Table 1:** Structures of investigated molecular fluorescent sensors for photopolymerization processes.

**Structures of the Derivatives of 2-amino-4,6-diphenyl-pyridine-3-carbonitrile**					
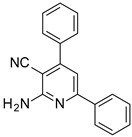		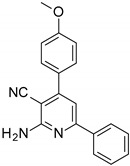		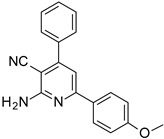	
S1		S2		S3	
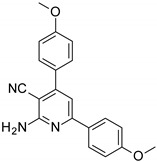		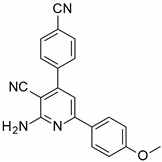		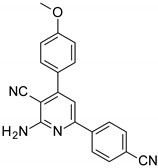	
S4		S5		S6	
**Reference Molecular Fluorescent Sensors**					
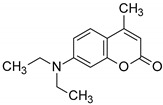			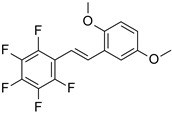		
C1			25ST		

**Table 2:** Spectral characteristics of the 2-amino-4,6-diphenyl-pyridine-3-carbonitrile derivatives studied.

Compound	Concentration [mol·dm^−3^]	λ_ab-max_ [nm]	ε@λ_ab-max_ [dm^3^·mol^−1^·cm^−1^]	ε@320 nm [dm^3^·mol^−1^·cm^−1^]	λ_fl_ @λ_ex-320nm_ [nm]	Intensity @λ_fl-max_ [a. u.]
S1	7.38 × 10^−5^	349	15,890	4835	418	19,890
S2	6.84 × 10^−5^	354	12,750	6874	407	15,000
S3	7.77 × 10^−5^	357	13,780	4532	409	14,580
S4	7.37 × 10^−5^	357	14,830	7068	404	22,010
S5	7.18 × 10^−5^	364	13,990	4668	448	9610
S6	6.99 × 10^−5^	363	12,460	6473	432	27,420

**Table 3:** Spectroscopic data of 2-amino-4,6-diphenyl-pyridine-3-carbonitrile derivatives during the photopolymerization processes.

**Sensor**	**λ_max-BEFORE_** **[nm]**	**Intensity** **@λ_max-BEFORE_** **[a.u.]**	**λ_max-AFTER POL_ [nm]**	**Intensity** **@λ_max-AFTER_** **[a.u.]**	**|ΔI_max_|** **[a.u.]**	**ΔI_max_^a^** **[%]**	**Δλ_max_** **[nm]**	**Relative Sensitivity ^b^**
Free-radical photopolymerization process of TMPTMA under 320 nm								
S1	413	35,770	404	29,800	5970	17	9	2.15
S2	407	36,530	401	27,220	9310	25	6	1.56
S3	413	63,550	405	54,390	9160	14	8	1.55
S4	406	48,980	402	37,910	11,070	23	4	1.32
S5	440	16,630	427	15,180	1450	9	13	2.76
S6	426	39,030	420	28,200	10,830	28	6	1.77
C1-ref.	423	22,900	418	12,920	9980	44	5	1.00
**Sensor**	**λ_max-BEFORE_** **[nm]**	**Intensity** **λ_max-BEFORE_** **[a.u.]**	**λ_max-AFTER_** **[nm]**	**Intensity** **@λ_max-AFTER_** **[a.u.]**	**|ΔI_max_|** **[a.u.]**	**ΔI_max_^a^** **[%]**	**Δλ_max_** **[nm]**	**Relative Sensitivity ^b^**
Thiol-ene photopolymerization process of TMPTMA/MERCAPTO under 320 nm								
S1	413	21,950	405	24,290	2340	11	8	0.53
S2	409	38,060	403	42,850	4790	13	6	1.39
S3	414	33,000	406	39,340	6340	19	8	1.49
S4	407	56,550	403	65,970	9420	17	4	1.12
S5	442	19,030	428	25,280	6250	33	14	2.93
S6	428	50,860	423	51,330	470	1	5	1.44
C1-ref.	426	26,910	421	41,190	14,280	53	5	1.00
**Sensor**	**λ_max-BEFORE_** **[nm]**	**Intensity** **λ_max-BEFORE_** **[a.u.]**	**λ_max-AFTER_** **[nm]**	**Intensity** **@λ_max-AFTER_** **[a.u.]**	**|ΔI_max_|** **[a.u.]**	**ΔI_max_^a^** **[%]**	**Δλ_max_** **[nm]**	
Cationic photopolymerization process of TEGDVE under 320 nm								
S1	420	11,760	423	40,050	28,290	241	3	
S2	415	12,580	426	17,210	4630	37	11	
S3	417	12,220	447	38,940	26,720	219	30	
S4	412	16,110	442	26,270	10,160	63	30	
S5	450	16,540	463	54,140	37,600	227	13	
S6	438	11,910	441	23,090	11,180	94	3	
25ST-ref.	459	11,180	440	9790	1390	12	19	

^a^ Changes in fluorescence intensity expressed as a percentage in relation to the initial value before polymerization. ^b^ Relative sensitivity (SEN) S_rel_= [(R_max_ − R_0_)/R_0_]/[[(R_max-ref_ − R_0-ref_)/R_0-ref_]; where R_max_—the ratio after polymerization for fluorescent sensor, R_0_ the initial ratio before polymerization for fluorescent sensor; R_max-ref_—the ratio after polymerization for Coumarin 1, R_0-ref_ the initial ratio before polymerization for Coumarin 1.

**Table 4:** Functional group conversions of vinyl monomer for TEGDVE and epoxy monomer for CADE and using photoinitiating system based on diphenyliodonium hexafluorophosphate (HIP wt 1%) and 2-amino-4,6-diphenyl-pyridine-3-carbonitrile derivatives in the role of co-initiator at 365 nm exposure.

Fluorescent Sensor/ Co-initiator	ε @λ_ab-365_ [dm^3^·mol^−1^·cm^−1^]	E_ox_^1/2^ [mV]	E_00_ [eV]	ΔG_et_ [eV]	Conversion of Double Bonds in TEGDVE @365 nm (95 mW/cm^2^)	Conversion of CADE Epoxy Monomer @365 nm (190 mW/cm^2^)
S1	8410	1584	3.22	−0.92	83	50
S2	10,330	1635	3.10	−0.74	83	71
S3	12,970	1521	3.06	−0.82	82	78
S4	13,820	1602	3.09	−0.77	83	71
S5	13,990	1157	3.06	−1.18	85	78
S6	12,410	1664	3.10	−0.71	82	72

E_ox_^1/2^—the electrochemically determined oxidation half-wave potentials (vs. Ag/AgCl) of the photosensitizers (the electron donor); E_red_^1/2^—the electrochemically determined reduction half-wave potentials (vs. Ag/AgCl) of the diphenyliodonium hexafluorophosphate (the electron acceptor)—E_red_^1/2^_HIP_ = −0.68V vs. SCE (−0.72V vs. Ag/AgCl); E_00_—the excitation energy of the photosensitizer, which is referred to as singlet excitation energy; ΔG_et_—the enthalpy of free electron transfer (calculated from the equation number 1).

**Table 5:** Functional group conversions of acrylate monomer for TMPTA, and thiol and methacrylate monomers for thiol-ene photopolymerization process, using photoinitiating system based on diphenyliodonium hexafluorophosphate (HIP wt 1%) and 2-amino-4,6-diphenyl-pyridine-3-carbonitrile derivatives in the role of co-initiator at 365 nm exposure.

Fluorescent Sensor/ Co-Initiator	Free-Radical Photopolymerization	Thiol-ene Photopolymerization	
	Conversion of Double Bonds in TMPTA @365 nm (95 mW/cm^2^)	Conversion of Double Bonds in TMPTMA in thiol-ene Polymerization @365 nm (95 mW/cm^2^)	Conversion of thiol Bonds in Mercapto in thiol-ene Polymerization @365 nm (95 mW/cm^2^)
S1	44	93	45
S2	46	73	35
S3	25	93	46
S4	49	92	45
S5	30	95	45
S6	47	89	44

## Supplementary Materials

The following are available online at https://www.mdpi.com/1424-8220/19/7/1668/s1, the synthesis of S1–S6, the procedures are fully presented in supporting information.
