# Supplementary material for: The Applicability of 2-amino-4,6-diphenyl-pyridine-3-carbonitrile Sensors for Monitoring Different Types of Photopolymerization Processes and Acceleration of Cationic and Free-Radical Photopolymerization Under Near UV Light
Source: Sensors (Basel). 2019 Apr 8;19(7):1668. doi: 10.3390/s19071668 (PMC6480573; doi:10.3390/s19071668)
Supplement: Supplementary file 1 [file sensors-19-01668-s001.pdf]

Supplementary material

The applicability of 2-amino-4,6-diphenyl-pyridine-3-carbonitrile sensors for monitoring different types of photopolymerization processes and acceleration of cationic and free-radical photopolymerization under near UV light

Joanna Ortyl[1,2 *], Paweł Fiedor[1], Anna Chachaj-Brekiesz[3], Maciej Pilch[1], Emilia Hola[1], Mariusz Galek[2]

[1] Cracow University of Technology, Faculty of Chemical Engineering and Technology, Warszawska 24, 31-155 Kraków, Poland

[2] Photo HiTech Ltd., Bobrzyńskiego 14, 30-348 Kraków, Poland

[3] Jagiellonian University, Faculty of Chemistry, Gronostajowa 2, 30-387 Kraków, Poland

Table of contents:

1. Preparation and characterization of 2-amino-4,6-diphenyl-pyridine-3-carbonitrile derivatives ……………………………………………………………………..…...**S2**

1.1. Materials and methods ………………………………………………..………...**S2**

1.2. Synthesis of 2-benzylidenepropanedinitrile derivatives ……………………...…..**S2**

1.3. Synthesis of 2-amino-4,6-diphenylpyridine-3-carbonitrile derivatives …...………**S3**

2. NMR spectra of 2-amino-4,6-diphenylpyridine-3-carbonitrile derivatives ..…..……**S5**

3. Materials for photopolymerization processes ……………………………………..… **S8**

4. References ………………………………………………………………………..…**S8**

[*]Corresponding author:
e-mail: jortyl@chemia.pk.edu.pl (Joanna Ortyl)

1. *Preparation and characterization of 2-amino-4,6-diphenyl-pyridine-3-carbonitrile derivatives*

Derivatives of 2-amino-4,6-diphenylpyridine-3-carbonitrile were synthesized by modified protocol by Kambe et al. [48]. This procedure involved condensation reaction between earlier synthesized 2-benzylidenepropanedinitrile derivative and commercially available acetophenone.

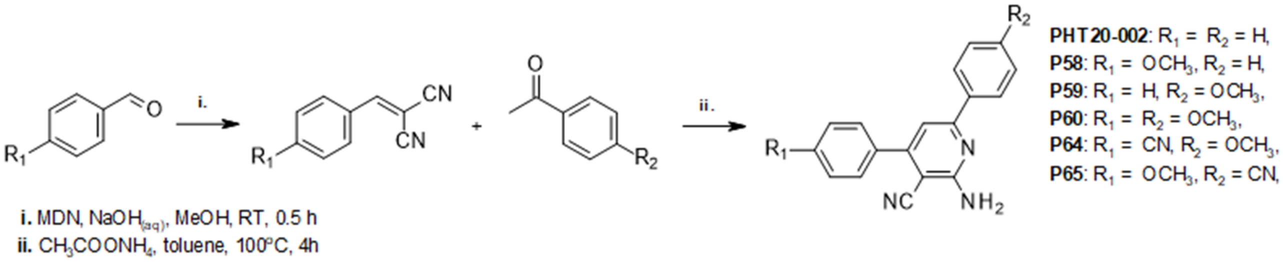

1.1. *Materials and methods*

Reagents for synthesis were purchased from Sigma-Aldrich or Alfa Aesar and used without further purification.

Structure and purity of obtained products were confirmed by NMR and LC-MS analysis. ^{1}H NMR spectra were recorded in DMSO-D_6 on Avance III HD 400 MHz (Bruker) spectrometer. Chemical shifts are reported in parts per million (δ) and referenced to residual protonated solvent peak (δ = 2.50 ppm). LC-MS analyses were obtained on Shimadzu LCMS-2020 with ESI ionization method. Acetonitrile was used as eluent.

1.2. *Synthesis of 2-benzylidenepropanedinitrile derivatives*

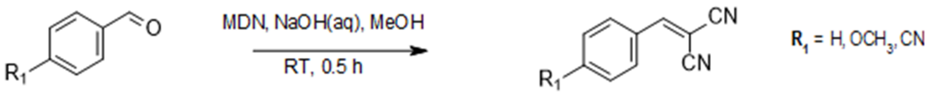

The solution of sodium hydroxide (0.5 mmol, 20 mg) in water (5.0 cm^3) was added dropwise to mixed solution of appropriate benzaldehyde derivative (10.0 mmol) and malononitrile (12.0 mmol, 793 mg) in methanol (30.0 cm^3). After 30 minutes of stirring the resulting crystal-like precipitate was filtered under reduced pressure and dried under vacuum.

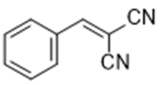

2-benzylidenepropanedinitrile,
10.00 g, yield: 65%,
^{1}H NMR (400 MHz, DMSO) δ 8.56 (s, 1H), 7.99 – 7.92 (m, 2H), 7.73 – 7.66 (m, 1H), 7.66 – 7.59 (m, 2H).

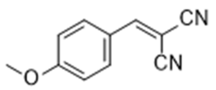

2-[(4-methoxyphenyl)methylene]propanedinitrile,
15.76 g, yield: 86%,
^{1}H NMR (400 MHz, DMSO) δ 8.39 (s, 1H), 8.00 – 7.94 (m, 2H), 7.22 – 7.15 (m, 2H), 3.88 (s, 3H).

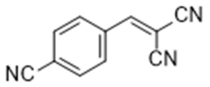

2-[(4-cyanophenyl)methylene]propanedinitrile,
16.10 g, yield: 90%,
^{1}H NMR (400 MHz, DMSO) δ 8.65 (s, 1H), 8.12 – 8.07 (m, 2H), 8.07 – 8.02 (m, 2H).

1.3. Synthesis of 2-amino-4,6-diphenylpyridine-3-carbonitrile derivatives

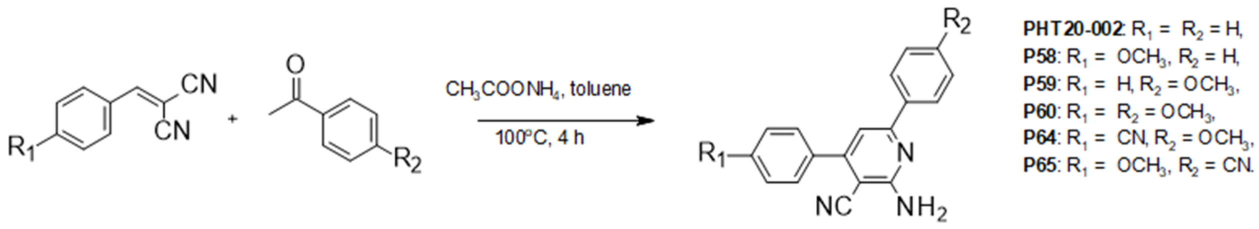

Ammonium acetate (7.10 mmol, 547 mg) was added to mixed solution of appropriate derivatives of acetophenone (6.50 mmol) and 2-benzylidenepropanedinitrile (6.50 mmol) in toluene (10.0 cm^3). Resulting mixture was heated for 4 hours in temperature 100°C. Obtained solution was washed with water and saline, dried over anhydrous magnesium sulphate and concentrated under reduced pressure. Product with satisfying purity was obtained after crystallization from methanol.

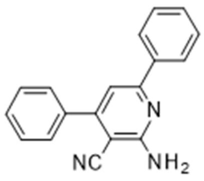

2-amino-4,6-diphenylpyridine-3-carbonitrile (PHT20-002);
0.698 g Yield: 33%;
purity (LC): >99%;
^{1}H NMR (400 MHz, DMSO) δ 8.17 – 8.10 (m, 2H), 7.72 – 7.65 (m, 2H), 7.60 – 7.53 (m, 3H), 7.52 – 7.46 (m, 3H), 7.28 (s, 1H), 7.02 (brs, 2H);
MS (ESI) m/z(%): 272 ([M+H]$^+$, 28%), 313 ([M+CH_3CN]$^+$, 100%).

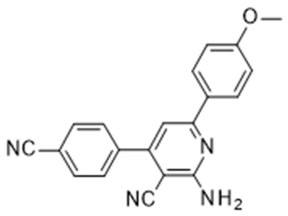

2-amino-4-(4-cyanophenyl)-6-(4-methoxyphenyl)pyridine-3-carbonitrile (P64);
0.730 g, yield: 30%;
purity (LC): 95%;
^{1}H NMR (400 MHz, DMSO) δ 8.15 – 8.09 (m, 2H), 8.07 – 8.01 (m, 2H), 7.90 – 7.84 (m, 2H), 7.27 (s, 1H), 7.09 – 7.01 (m, 4H), 3.82 (s, 3H);
MS (ESI) m/z(%): 327 ([M+H]$^+$, 19%), 368 ([M+CH_3CN]$^+$, 100%).

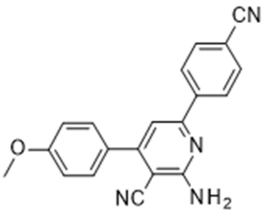

2-amino-6-(4-cyanophenyl)-4-(4-methoxyphenyl)pyridine-3-carbonitrile (P65);
0.370 g, yield: 15%;
purity (LC): 94%;
^{1}H NMR (400 MHz, DMSO) δ 8.34 – 8.28 (m, 2H), 8.00 – 7.93 (m, 2H), 7.71 – 7.65 (m, 2H), 7.38 (s, 1H), 7.14 – 7.10 (m, 2H), 7.08 (brs, 2H), 3.84 (s, 3H);
MS (ESI) m/z(%): 327 ([M+H]$^+$,10%), 368 ([M+CH_3CN]$^+$, 100%)

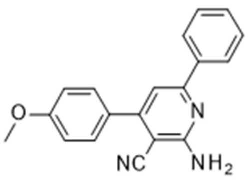

2-amino-4-(4-methoxyphenyl)-6-phenylpyridine-3-carbonitrile (P58);
0.630 g, yield: 27%;
purity (LC): >99%;
^{1}H NMR (400 MHz, DMSO) δ 8.15 – 8.09 (m, 2H), 7.70 – 7.63 (m, 2H), 7.53 – 7.44 (m, 3H), 7.25 (s, 1H), 7.14 – 7.08 (m, 2H), 6.95 (brs, 2H), 3.84 (s, 3H);
MS (ESI) m/z(%): 302 ([M+H]$^+$,64%), 343 ([M+CH_3CN]$^+$, 100%).

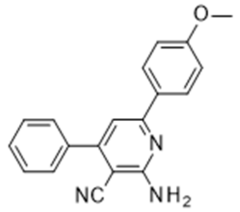

2-amino-6-(4-methoxyphenyl)-4-phenylpyridine-3-carbonitrile (P59);
0.790 g, yield: 34%;
purity (LC): >99%;
^{1}H NMR (400 MHz, DMSO) δ 8.15 – 8.08 (m, 2H), 7.70 – 7.63 (m, 2H), 7.58 – 7.50 (m, 3H), 7.22 (s, 1H), 7.07 – 7.00 (m, 2H), 6.94 (brs, 2H), 3.82 (s, 3H);
MS (ESI) m/z(%): 302 ([M+H]$^+$,62%), 343 ([M+CH_3CN]$^+$, 100%).

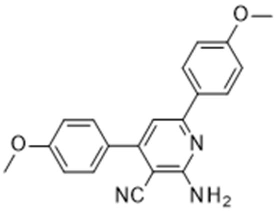

2-amino-4,6-bis(4-methoxyphenyl)pyridine-3-carbonitrile (P60);
0.800 g, yield: 32%;
purity (LC): >99%;
^{1}H NMR (400 MHz, DMSO) δ 8.14 – 8.06 (m, 2H), 7.68 – 7.61 (m, 2H), 7.19 (s, 1H), 7.14 – 7.07 (m, 2H), 7.06 – 7.00 (m, 2H), 6.87 (brs, 2H), 3.84 (s, 3H), 3.82 (s, 3H);
MS (ESI) m/z(%): 332 ([M+H]$^+$,74%), 373 ([M+CH_3CN]$^+$, 100%).

2. ^{1}HNMR spectra of 2-amino-4,6-diphenylpyridine-3-carbonitrile derivatives

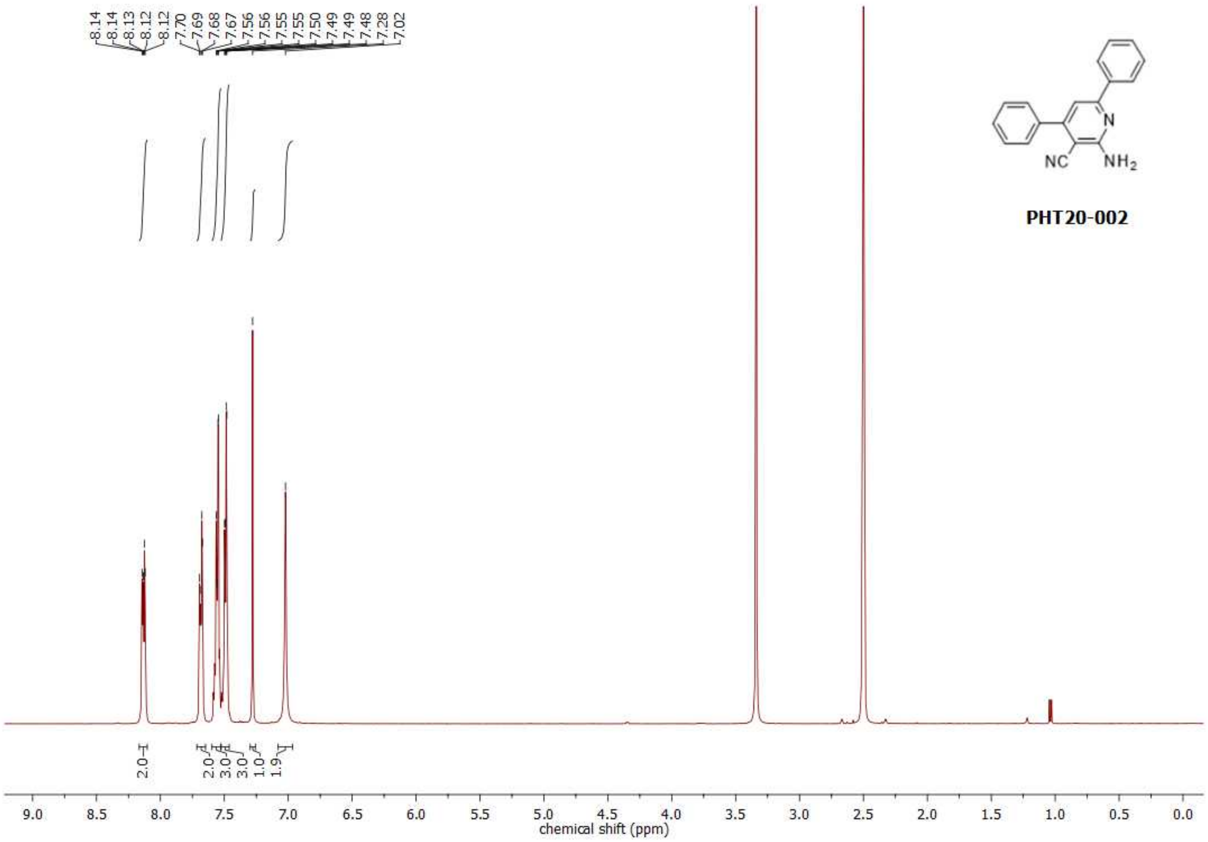

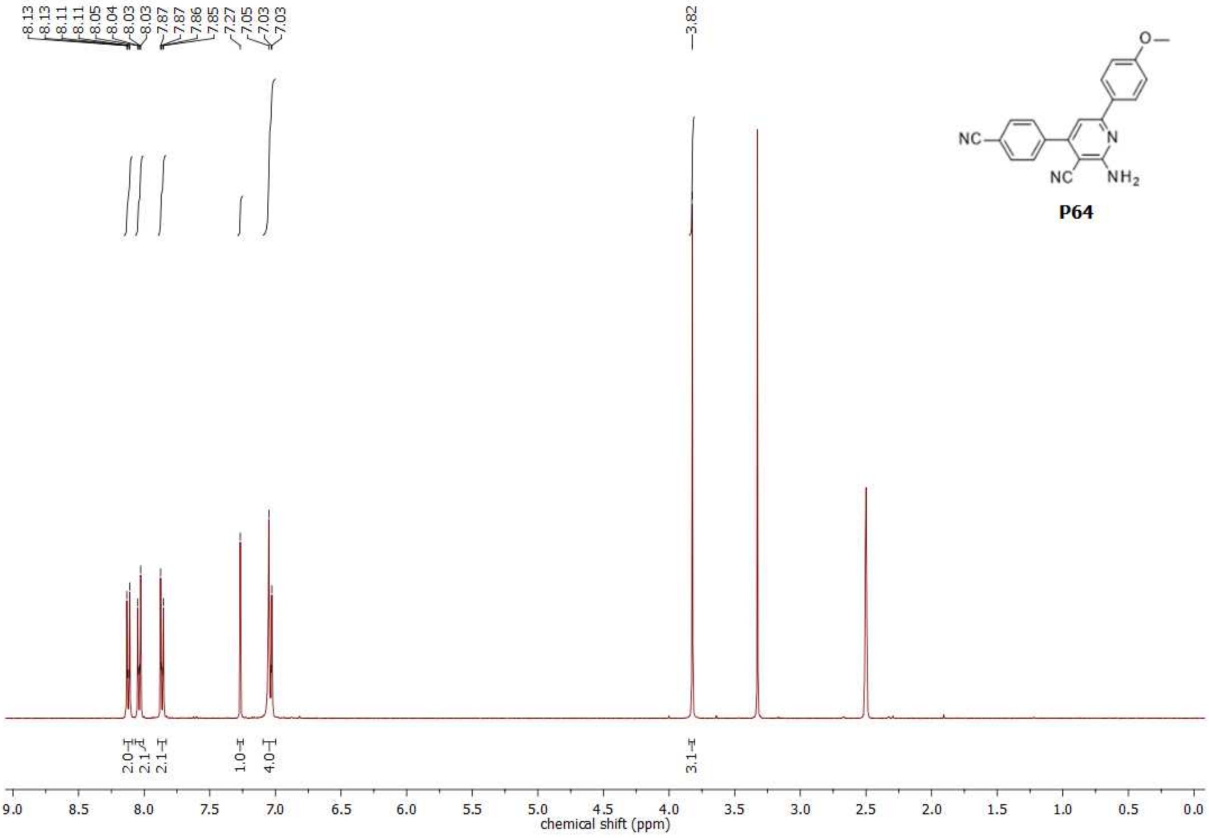

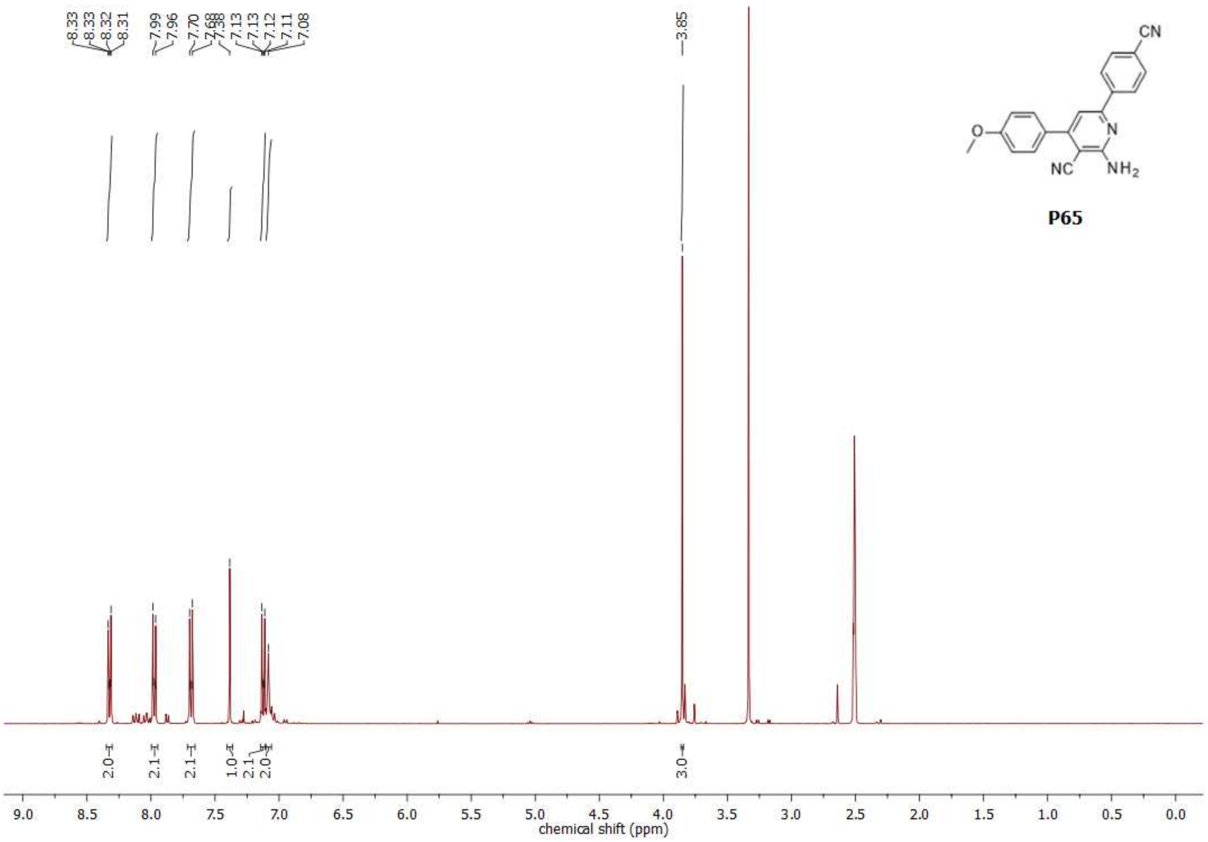

8.33
8.33
8.32
8.31
7.99
7.96
7.70
7.68
7.38
7.13
7.13
7.12
7.11
7.08
3.85
CN
O
N
NC
NH2
P65
2.0
2.1
2.1
1.0
2.1
2.0
3.0
9.0 8.5 8.0 7.5 7.0 6.5 6.0 5.5 5.0 4.5 4.0 3.5 3.0 2.5 2.0 1.5 1.0 0.5 0.0
chemical shift (ppm)

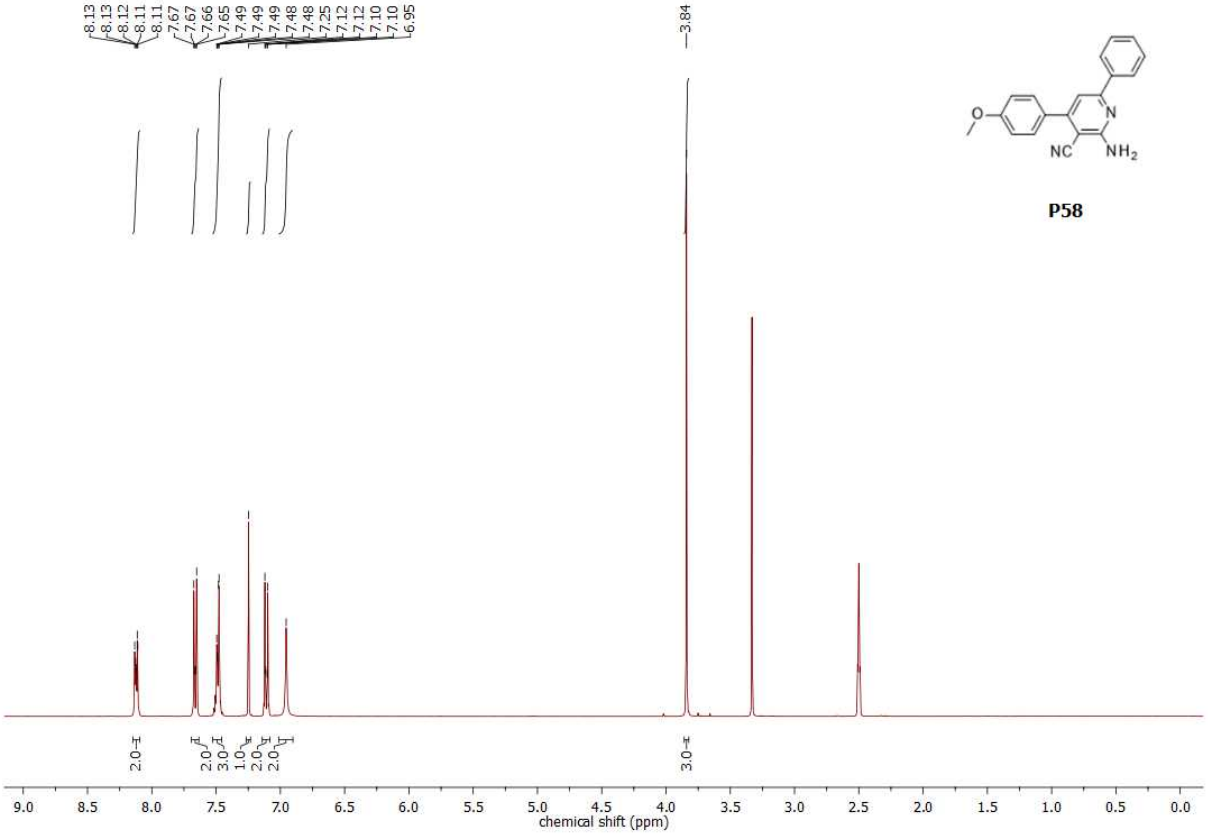

8.13
8.13
8.12
8.11
8.11
7.67
7.67
7.66
7.65
7.49
7.49
7.49
7.48
7.48
7.25
7.12
7.12
7.10
7.10
6.95
3.84
O
N
NC
NH2
P58
2.0
2.0
3.0
1.0
2.0
2.0
3.0
9.0 8.5 8.0 7.5 7.0 6.5 6.0 5.5 5.0 4.5 4.0 3.5 3.0 2.5 2.0 1.5 1.0 0.5 0.0
chemical shift (ppm)

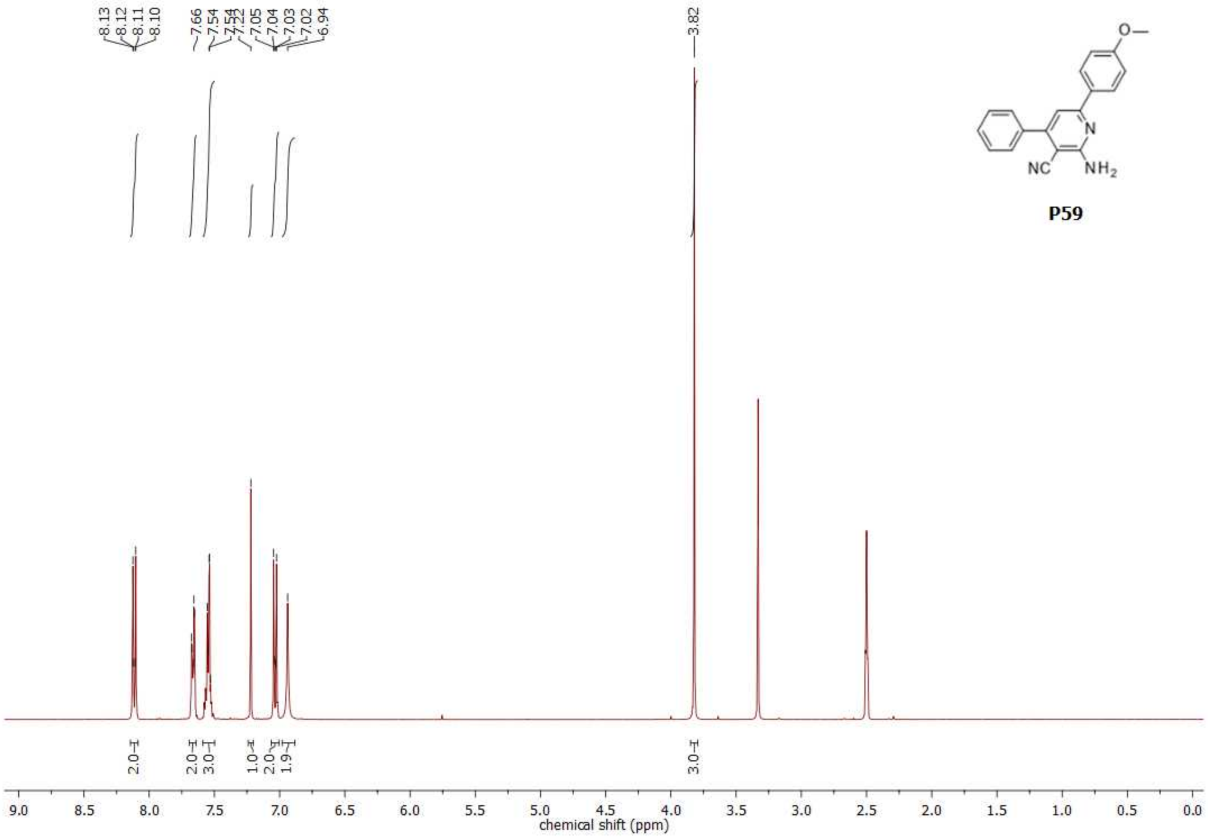

P59

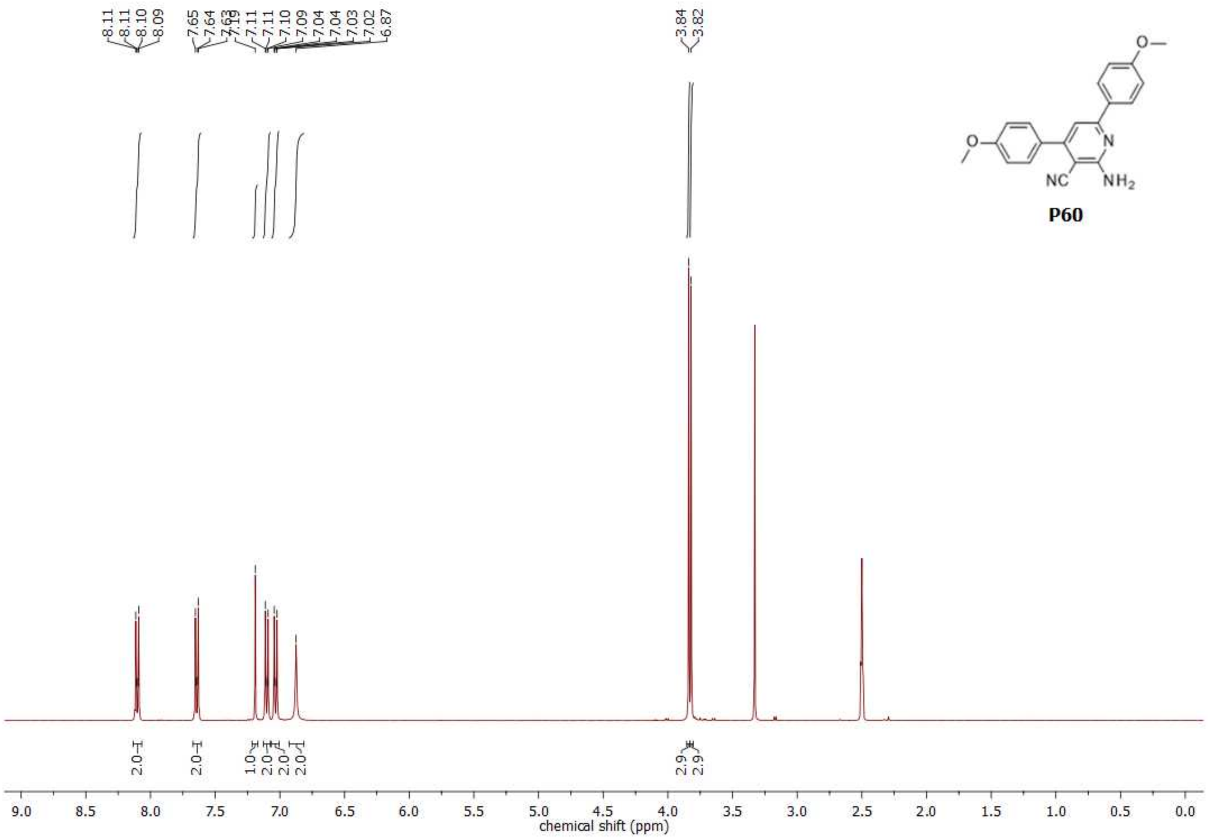

P60

3. Materials for photopolymerization processes

3.1. Cationic photopolymerization under UV light with λ_{max} =320 nm and under λ_{max} =365 nm respectively.

3,4-epoxycyclohexylmethyl 3,4-epoxycyclohexanecarboxylate (CADE, from Allnex) as a cycloaliphatic epoxide monomer was used for the compositions cured by ring opening cationic photopolymerization. Triethylene glycol divinyl ether (TEGDVE, from Sigma Aldrich) was applied as a model vinyl ether monomer for cationic chain photopolymerization. For the role of cationic photoinitiator the diphenyliodonium hexafluorophosphate (HIP, from Alfa Aesar) was used under UV light with λ_{max} =320 nm. The bimolecular photoinitiating systems based on diphenyliodonium hexafluorophosphate (HIP) and 2-amino-4,6-diphenyl-pyridine-3-carbonitrile derivatives were used during the photopolymerization processes under UV light with λ_{max} =365 nm.

3.2. Free-radical photopolymerization under UV light with λ_{max} =320 nm.

Trimethylopropane triacrylate (TMPTA, from Sigma Aldrich) and 2,2-dimethoxy-2-phenylacetophenone (DMPA, from Sigma Aldrich), were employed as an methacrylate monomer and a free-radical photoinitiator 2,2-dimethoxy-2-phenylacetophenone (DMPA, from Sigma Aldrich) for the compositions polymerized by a free-radical mechanism for FPT experiments under UV light with λ_{max} =320 nm.

3.3. Free-radical photopolymerization under UV light with λ_{max} =365 nm.

For the compositions polymerized by a free-radical mechanism under UV light with λ_{max} =365 nm were used trimethylopropane triacrylate (TMPTA, from Sigma Aldrich) as a model acrylate monomer and the bimolecular systems based on diphenyliodonium hexafluorophosphate (HIP) and 2-amino-4,6-diphenyl-pyridine-3-carbonitrile derivatives as photoinitiators.

3.4. Thiol-ene photopolymerization under UV light with λ_{max} =320 nm.

Moreover, trimethylopropane trimethacrylate (TMPTMA, from Sigma Aldrich) and trimethylopropane tris(3-mercaptopropionate) (MERCAPTO, from Sigma Aldrich) were utilized as a methacrylate and thiol monomers for the compositions polymerized by the thiol-ene mechanism. 2,2-dimethoxy-2-phenylacetophenone (DMPA, from Sigma Aldrich) was applied as the photoinitiator in the thiol-ene polymerization process for FPT experiments under UV light with λ_{max} =320 nm.

3.5. Thiol-ene photopolymerization under UV light with λ_{max} =365 nm.

Respectively, trimethylopropane trimethacrylate (TMPTMA, from Sigma Aldrich) and trimethylopropane tris(3-mercaptopropionate) (MERCAPTO, from Sigma Aldrich) were applied as the monomers for thiol-ene process of photopolymerization. For the role of UV -long wavelength photoinitiators were used diphenyliodonium hexafluorophosphate (HIP) and 2-amino-4,6-diphenyl-pyridine-3-carbonitrile derivatives.
Structures of the monomers are shown in Table 1.

Table 1. Chemical structures of the monomers and iodonium salt.

**One component photoinitiators**		
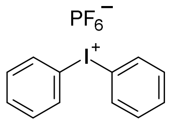 HIP – one component cationic photoinitiator	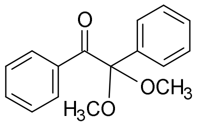 DMPA – one component free-radical photoinitiator	
**Monomers**		
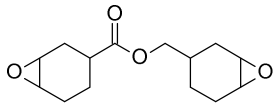 CADE	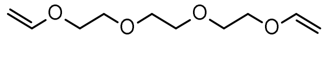 TEGDVE	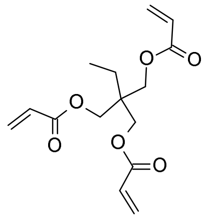 TMPTA
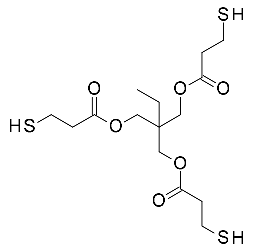 MERCAPTO	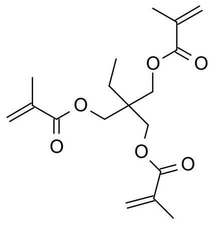 TMPTMA	

4. ***References:***

[48] Kambe, S., Saito, K., Sakurai, A., Midorikawa, H., A simple method for the preparation of 2-amino-4-aryl-3-cyanopyridines by condensation of malononitrile with aromatic aldehydes and alkyl ketones in the presence of ammonium acetate, Synthesis 1980, 366-368
